# A microgrid deployment framework to support drayage electrification

**DOI:** 10.1016/j.isci.2025.113804

**Published:** 2025-10-16

**Authors:** Joseph N.E. Lucero, Ruixiao Sun, Brandon A. Miller, Simona Onori, Vivek A. Sujan

**Affiliations:** 1 Department of Chemistry, Stanford University, Stanford, CA 94305, USA; 2 Buildings and Transportation Science Division, Oak Ridge National Laboratory, Oak Ridge, TN 37831, USA; 3 Computational Sciences and Engineering Division, Oak Ridge National Laboratory, Oak Ridge, TN 37831, USA; 4 Department of Energy Science and Engineering, Stanford University, Stanford, CA 94305, USA; 5 Applied Energy Division, SLAC National Accelerator Laboratory, Menlo Park, CA 94025, USA

**Keywords:** Energy policy, Engineering, Electrical engineering

## Abstract

The electrification of heavy-duty commercial vehicles (HDCVs) is key to enhancing air quality and reducing urban air pollution, but it also imposes significant demands on an electric grid not designed for such high loads. Without complementary infrastructure, electrification may yield limited air quality improvements. This article explores the critical role of microgrids—integrating solar photovoltaics and battery storage—in supporting HDCV electrification. We present an integrated framework to identify viable microgrid sites in a given region, estimate deployment costs, and optimize power use to reduce dependence on the grid. As a case study, we apply the framework to the region around the Port of Savannah, GA, USA, demonstrating how targeted microgrid deployment can enhance grid capacity, improve energy resiliency, and support electrified freight transport.

## Highlights

• Microgrids can close grid power gaps from heavy-duty EV fleet charging, boosting resilience
• Microgrids, especially with LFP batteries, can significantly reduce electrification costs
• The microgrid framework is modular and data-driven, enabling siting analysis across the US

## Graphical abstract

**Figure:**
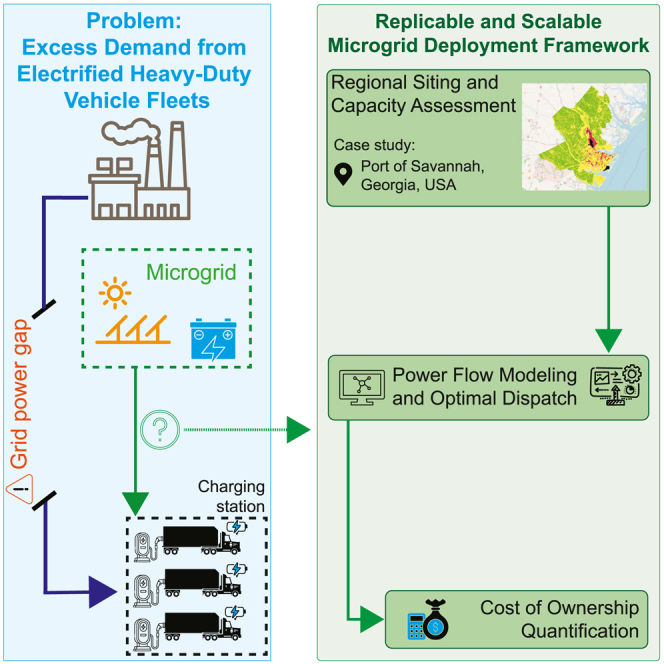

## Introduction

The transportation sector is undergoing a significant transformation with the electrification of heavy-duty commercial vehicles (HDCVs), particularly in drayage applications around intermodal hubs such as maritime ports.^1^^,^^2^ These diesel-powered vehicles, essential for “first-mile” delivery between ports and distribution centers, contribute substantially to air pollution in urban areas. As countries work toward energy resiliency, transitioning to electric drayage vehicles is becoming increasingly important.^3^

However, the widespread adoption of electric HDCVs faces challenges related to electricity demand and grid capacity. The current grid, designed for residential and light commercial needs, may struggle to support the high energy requirements of charging infrastructure for large vehicles. Microgrids, which integrate alternative energy sources and energy storage systems, can alleviate this strain by supplementing grid capacity without requiring costly upgrades. These localized solutions thus enhance grid resilience and support the transition to an electrified transportation system.

This work focuses on the deployment of a microgrid incorporating solar power and lithium-ion battery (LIB) energy storage to meet the energy demands of an electrified HDCV fleet. Solar energy, combined with battery storage, offers alternative power sources that reduce reliance on fossil fuels and stabilize energy supply during peak demand periods. The use of LIBs is highlighted for their efficiency, longevity, and decreasing costs, making them ideal for grid applications.^4^^,^^5^

The concept of HDCV electrification, particularly in drayage applications, has been actively explored in recent years. In particular, studies have highlighted the potential of electric trucks to improve air quality significantly, especially near port areas and surrounding urban regions where this is a major concern.^6^^,^^7^ This research indicates that the electrification of drayage trucks operating at the port can lead to substantial reductions in harmful diesel-related pollutants. Therefore, although studies show that investments in infrastructure and vehicles will be initially costly, the long-term benefits in terms of fuel savings, maintenance costs, improved operational reliability, and air quality benefits are expected to be substantial^7^^,^^8^^,^^9^^,^^10^

While there are clear benefits to the electrification of HDCVs, it also creates significant electricity demand surges at depots and intermodal hubs such as ports, raising concerns about grid reliability and infrastructure readiness. Studies assessing the impact of large-scale electrification have found that, while many substations can accommodate fleet electrification with minimal upgrades, for some high-density freight corridors and regions where electricity networks are already strained, this added burden could lead to voltage instability, higher operational costs for the fleet, as well as the need for expensive grid reinforcements.^11^

Efforts to manage these grid impacts have explored coordinated charging strategies, demand response programs, and vehicle-to-grid integration.^12^ While these approaches can mitigate localized demand spikes, they do not fully eliminate the need for infrastructure expansion in high-density freight corridors. Some studies suggest that deploying microgrids at fleet depots and port terminals could reduce dependence on the broader grid while supporting the scalability of fleet electrification efforts^13^; however, systematic methodologies to determine optimal microgrid siting in these environments remain limited.

Integrating alternative energy sources into microgrids has been recognized as a cost-effective way to supplement grid power and enhance energy resilience. Research on microgrid deployment for commercial and industrial applications suggests that solar and battery storage systems can effectively smooth out power fluctuations and reduce reliance on expensive peak-hour electricity from the grid^14^^,^^15^^,^^16^; however, designing microgrids for HDCV fleets introduces unique challenges as energy consumption patterns are highly variable and charging events tend to cluster within specific time frames.^15^ This clustering of charging events increases peak loads and operational costs for fleet operators. Studies on charging optimization have shown that managed charging schedules, route-based energy planning, and depot-level forecasting can significantly reduce peak demand without compromising operational efficiency.^7^^,^^12^

Emerging technologies such as vehicle-to-grid (V2G) and vehicle-to-building (V2B) integration have also been proposed as demand flexibility solutions, allowing trucks to temporarily supply power back to microgrids or the broader grid during idle periods.^11^ While these approaches offer economic and resilience benefits, their effectiveness is contingent on battery degradation rates, charging infrastructure compatibility, and regulatory frameworks.

Building on prior work that developed regional electrification roadmaps for HDCVs (OR-AGENT),^17^ the present study enhances the existing microgrid siting procedure by proposing an integrated, site-specific framework for microgrid deployment to support HDCV electrification. Specifically, it introduces three innovations: (1) a siting pipeline that combines ORNL’s “Oak Ridge Siting Analysis for Generation Expansion” (OR-SAGE) tool with NREL’s “Renewable Energy Potential” (reV) model to identify and assess the potential of viable microgrid sites, (2) a power distribution optimization model that maximizes air quality improvements through coordinated use of solar, battery, and grid resources, and (3) a total cost of ownership (TCO) analysis that quantifies deployment costs from the perspective of the HDCV fleet operator. We apply this framework to the Port of Savannah, GA, and its surrounding counties—Chatham, Jasper, Bryan, Liberty, and Effingham—as a representative case study. While OR-AGENT provides the routing information and total load demands of the fleet, this work operationalizes those outputs into actionable infrastructure planning. By incorporating grid constraints, site-level feasibility, and economic analysis, it bridges the gap between regional electrification planning and practical deployment of microgrids.

### Two problems with fleet electrification

We briefly review how the excess (electricity) load demand for a fleet of electrified HDCVs is estimated, how the corresponding increase in pollutant intensity from the grid is computed, as well as how excess load demand stresses the present grid infrastructure and how this potentially undermines potential air quality improvements, one of the primary benefits of fleet electrification.

#### Estimating the excess load demand and air quality improvements of fleet electrification

To estimate the excess load demand due to HDCV electrification, we leverage a recently introduced framework known as the “Optimal Regional Architecture Generation for Electrified National Transport” (OR-AGENT) from ORNL, developed in collaboration with the Ohio State University and Stanford University.^17^ This framework uses a bottom-up approach to generate deployment roadmaps for HDCV electrification. To this end, OR-AGENT uses a comprehensive, forward-looking, electric HDCV powertrain simulator that accounts for various factors such as driver behavior, aerodynamic load variation based on truck configuration, tire thermal dynamics, rolling resistance, and power consumption of auxiliary components such as the cabin HVAC compressor and battery thermal management system while the vehicle operates in the region.^18^

For each month of the year, OR-AGENT determines the different routes traversed by the trucks, the number of trucks in operation on those routes, and the trucks’ associated weight/class statistics for a given U.S. region by integrating a variety of data sources.^17^ Using vehicle statistics and route features, along with simulator-based estimates of per-truck energy use, the hourly energy requirement by the full fleet of electrified trucks is estimated (see Figure S1).

The electrification of fossil fuel–based transportation is expected to improve regional air quality; however, transitioning existing HDCV fleets from diesel to battery-electric powertrains, without the deployment of additional non-fossil fuel-based energy resources, requires the existing grid infrastructure to supply the excess energy demand entirely. Although this transition would still yield net air quality improvements relative to continued diesel operation, the reliance on a grid that is not pollutant-minimal constrains the magnitude of those gains. This is particularly true in the region of the Port of Savannah.^17^ We henceforth refer to the difference between the theoretically attainable air quality improvements, had the additional electricity been sourced from pollutant-minimal resources, and the realized improvements under the present grid mix as “unrealized air quality improvements.” These unrealized improvements can be estimated using an approach adapted from Sujan et al.,^17^^,^^19^ which integrates historical spatiotemporal data on pollutant intensity and electrical load demand across all U.S. counties to forecast the pollutant intensity associated with the excess load. Given historical electric load profiles, regional generation mixes, and the projected increase in demand from fleet electrification, this method enables the quantification of the hourly unrealized air quality improvements for any given region (see Figure S2).

#### Identifying grid capacity gaps and mitigating air quality improvements

**Figure 1:**
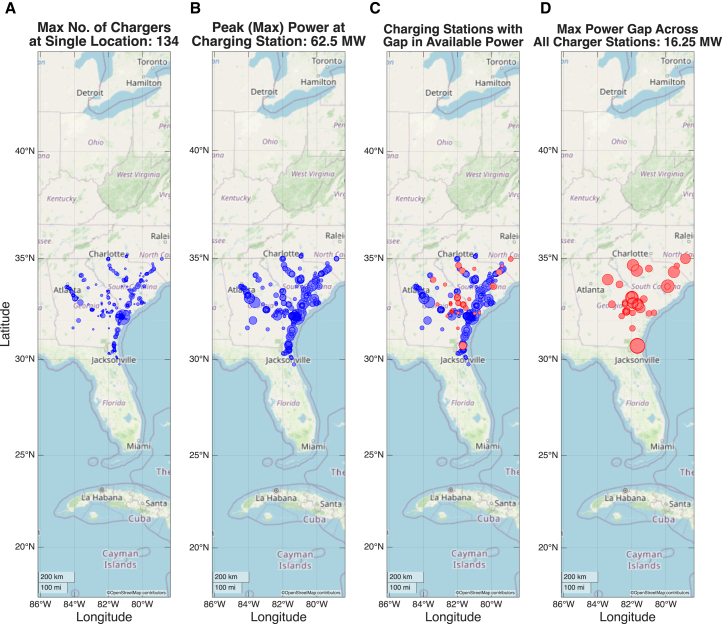
Grid power gap in the Port of Savannah region (A) Number of chargers required at each station, including destinations and the Port of Savannah, to meet demand. The largest point represents a maximum of 134 chargers. (B) Peak power demands at each charging station. The largest point corresponds to a peak demand of 62.5 MW. (C) Locations where the existing grid capacity is insufficient to support peak charging demand, marked in red. (D) Isolating subset of locations from (C) where peak charging power exceeds grid capacity, with the largest point indicating an excess of 16.25 MW above available capacity.

To enable the full electrification of all of the routes in the Port of Savannah region, either in-route stationary charging or electrified road systems must be implemented to extend the range of HDCVs.^20^ Here, we consider the scenario where stationary charging is allowed at both at the Port of Savannah as well as at the destinations of the HDCVs. We find that a certain number of chargers must be installed at each location to meet the demand, as seen in Figure 1A^19^. Moreover, the peak power of each location in any given hour may also be estimated (Figure 1B).

To assess whether the existing grid can support the added demand from an electrified HDCV fleet, we identify all substations within a 15-mile radius of major freight routes. Using ORNL’s proprietary data and models, we evaluate each substation’s peak load and estimate the additional load that can be accommodated before any grid bus experiences a reduction in capability. Comparing these capacity limits to the hourly energy requirements of the electrified fleet (Figure 1C), we find that, in certain areas, demand exceeds present grid capacity. Addressing this shortfall will require the deployment of additional energy resources.

Relying on additional fossil fuel-based energy resources to meet excess demand can erode the air quality benefits of HDCV electrification. Specifically, in the Port of Savannah region, electrifying ∼80% of the fleet 800 kWh trucks, while maintaining diesel trucks for the rest, would cover all existing routes; however, using fossil fuel-based energy for the excess demand of the electrified fleet would reduce the amount of pollutant intensity by only 37.6% compared to a fully diesel-based fleet (see Figure S3).^21^ Thus, the deployment of alternative energy resources is essential for greater pollutant intensity reductions.

### Using a microgrid to meet increased electricity demands

#### The alternative energy network

**Figure 2:**
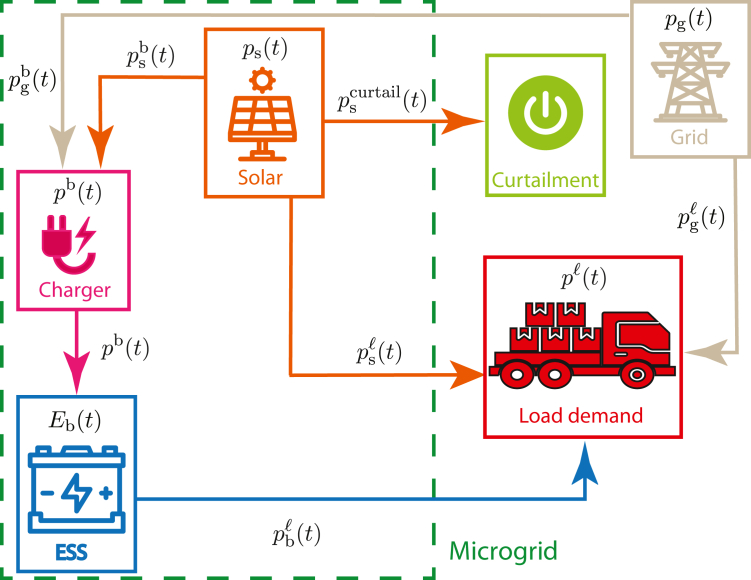
Schematic of energy network Colors indicate origin of power: orange lines from solar, blue lines from the battery (referred to as the energy storage system, or ESS, in the diagram), beige lines from the grid, and magenta lines from the charger. Curtailment acts as an energy sink for excess solar power.

We propose an *alternative energy network* that integrates a microgrid with solar (as the alternative energy source) and battery power to supplement the available grid power in meeting the excess load demand. In this context, we consider both existing and future fossil-fuel based resources as part of the “grid.” Figure 2 provides a schematic representation of this network, with arrows indicating the flow of energy between the network’s components.^22^^,^^23^ Table 1 summarizes the energy network variables and their interpretations.

**Table 1:** Energy network variables and their interpretation

Variable [Unit]	Interpretation
$p^{\ell }\left(t\right)$ [MW]	Excess load demand
*p*_s_(*t*)[MW]	Available solar power available
$p_{g}^{\ell }\left(t\right)$ [MW]	Power provided to load by grid
$p_{s}^{\ell }\left(t\right)$ [MW]	Power provided to load by solar
$p_{b}^{\ell }\left(t\right)$ [MW]	Power provided to load by battery
$p_{s}^{b}\left(t\right)$ [MW]	Power provided by solar to charge the battery
$p_{s}^{\text{curtail}}\left(t\right)$ [MW]	Curtailed solar power
*p*_g_(*t*)[MW]	Total power provided by the grid
*p*^b^(*t*)[MW]	Charging power to the battery
$p_{g}^{b}\left(t\right)$ [MW]	Power provided by grid to charge battery
*E*_b_(*t*)[MWh]	Battery energy remaining

In our notation, for a power quantity (units of MW) denoted as $p_{i}^{j}\left(t\right)$, the subscript *i*∈{*s*,*b*,*g*} denotes the network element origin of the power, which can be the solar, the battery, or the grid, and *j*∈{*b*,*l*} denotes the target network element, which can be the battery or the load. The variable *t* denotes time. We direct the reader to the supplemental information: Methods S1 – Section 2 for a mathematical formulation of the energy network.

#### Maximally improving air quality by orchestrating resources

We consider an energy network that relies solely on dispatching existing, fossil-fuel based, grid resources to meet the load demand as a *baseline network.* The corresponding baseline unrealized air quality improvements are calculated as the sum of the estimated hourly pollutant intensity. In contrast, the alternative energy network uses a mix of solar, battery, and grid power to meet the excess load demand. As such, the associated unrealized air quality improvements for this network are calculated based on hourly pollutant intensity scaled by the proportion of grid power dispatched relative to the baseline. Given the hourly available solar capacity *p*_s_(*t*) and the excess load demand profile $p^{\ell }\left(t\right)$, we formulate a mixed-integer linear program that determines the optimal power distribution from solar, battery, and the grid for the alternative energy network, at each hour of the year, which minimizes the unrealized air quality improvements, while continuing to meet the excess load demand. This approach extends previous work on the OR-AGENT framework^17^ by replacing the heuristic battery usage rules with a formally defined constrained optimization to maximize air quality improvements by explicitly considering energy storage constraints, alongside the time-varying solar availability. Furthermore, this approach is scalable and adaptable to different objectives and constraints. We direct the reader to supplemental information: Methods S1 – Section 2 for a formal presentation of the optimization problem.

### Microgrid siting framework

In this section, we describe how the potential for siting and generation of solar resources is modeled by integrating two existing frameworks: (1) the OR-SAGE tool from ORNL and (2) the reV model from NREL.^24^

#### Regional solar viability assessment

The assessment of solar viability is performed using OR-SAGE,^25^^,^^26^ a tool that leverages high-resolution geospatial data layers incorporating land-use restrictions based on federal guidelines, environmental/human impacts, and existing infrastructure. Each layer divides the region into 90 m × 90 m grid cells. Some layers, such as wetlands and open water, are known as decision layers, where each grid value directly indicates whether the corresponding area is suitable for siting a particular technology. In contrast, other layers, such as layers describing variations in slope, must be converted into a decision layer by using a filtering threshold on the data. Further details on the technology parameters and threshold values used for filtering in this analysis are provided in the supplemental information: Methods S1 – Section 3 (Table S2).

**Figure 3:**
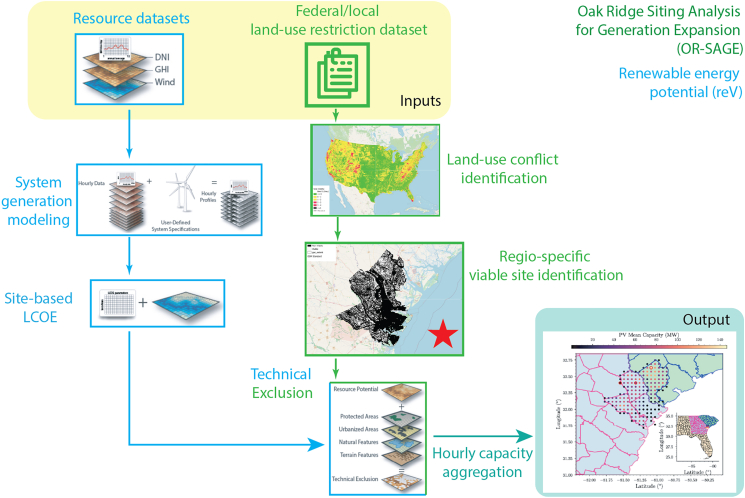
Schematic of the solar siting and hourly capacity estimation framework Elements marked in light blue represent components from the Renewable Energy Potential (reV) tool, while elements in green correspond to the Oak Ridge Siting Analysis for Generation Expansion (OR-SAGE) tool. The OR-SAGE tool yields a binary map that indicates whether a particular location can have solar deployed there. This map is integrated with the existing technical exclusion layers of reV (involving a site-based levelized cost of energy, or LCOE, analysis) to determine the solar availability across viable sites in the region. The image marked with a red star illustrates the binary map of non-viable subregions (shown in black) within the greater Port of Savannah area. The final output, after capacity aggregation, is the estimated solar availability for each site for every hour across a full calendar year. Figures for reV adapted from.^24^

Within each decision layer, a value of 0 indicates that a pixel is suitable for siting, and a value of 1 indicates a violation of the constraint specific to that layer. These data layers can then be overlayed to create a composite siting map, where the pixel value corresponds to the number of constituent data layers that conflict with a siting decision at the given location (see Figure S4). For this analysis, we focus on the region surrounding Chatham County, GA, where the Port of Savannah is located, and only parcels with no conflicts are considered viable for siting. Specifically, the three counties surrounding Chatham are also included in this analysis to encompass all available areas within the immediate surrounding region of the Port (see Figures S5 and S6). The section of the solar viability map corresponding to this region can be seen in the binary map in Figure 3 (highlighted with a red star).

#### Regional solar capacity availability

The calculations of technical potential and average capacity for the Port of Savannah region are performed using the various modules available in NREL’s reV tool,^24^ integrating the viability map created in the previous section. A schematic overview of these modules and how they are integrated with OR-SAGE is displayed in Figure 3.

The process begins with the selection of a region of interest (ROI). In reV, the ROI is defined by a series of grid points that are associated with traditional geographic coordinates. In the case of solar technical capacity, these grid points correspond to spatial data points within the National Solar Radiation Database (NSRDB), which measures three forms of solar radiation alongside a series of atmospheric and surface conditions during the 2019 calendar year for every hour, at an approximately 2 km resolution.^27^ Next, the generation module of reV is used to calculate capacity factor values––the ratio between the actual generation capacity of a site and its nameplate capacity––for a solar farm placed at each selected grid point in the NSRDB dataset (see Table S3). This is done for every hour in the chosen year, which results in a set of temporal capacity factor profiles for each farm.

**Figure 4:**
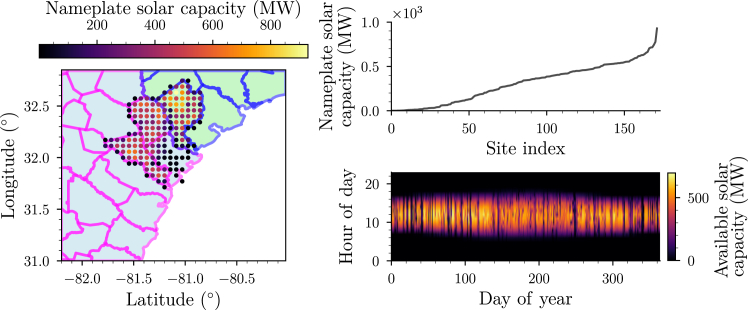
Viable solar sites in the region around the Port of Savannah (Left plot) Geographic locations of the viable sites colored by their nameplate capacity. (Right top plot) Nameplate solar capacity vs. site index. (Right bottom plot) Hourly available solar capacity for the largest nameplate solar capacity site.

Two processes follow using the reV model: (1) supply curve aggregation and (2) representative profile generation. Supply curve aggregation maps the high spatial resolution (90 m) site suitability data output from OR-SAGE to the low spatial resolution generation profiles of reV. To do this, each viable parcel (approximately 33 km^2^) is assigned a total technical capacity by multiplying the viable land area within the parcel by a scalar power density value of 36 MW/km^2^, the default value used in reV for fixed-tilt utility-scale PV plants.^24^ The resulting value is the nameplate capacity for a solar farm occupying all viable land in the parcel. For the Port of Savannah region considered, this aggregation identifies a set of 171 *viable solar sites*. The geographic locations of these sites, the nameplate solar capacity of each site, and the hourly solar availability for the highest nameplate solar capacity site are shown in Figure 4. By our convention, the sites are numbered in order of increasing nameplate solar capacity.

Representative profile generation produces hourly solar availability profiles, denoted by *p*_s_(*t*), for each site by combining the estimated capacity factor profile created in the generation module with the solar nameplate capacities computed in the supply curve aggregation step (see Figure S7).

### Estimating the costs of microgrid deployment

Having established the set of viable sites and the framework that determines how each element of the energy network is used if a microgrid is deployed at a given site, this section formulates the cost of deployment for the microgrid. Here, we consider the microgrid to be installed as a “front-of-the-meter” resource (i.e., utility-owned and feeds all charging depots within the region rather than installed to augment a specific charging depot). Thus, the capital costs of deployment are initially borne by the utility but recouped over the lifetime of the microgrid. The deployment of the microgrid affects (and can lower) the total cost of electricity charged to the fleet operator. A mathematical presentation of the costs and cost metrics described in this section is available in supplemental information: Methods S1 – Section 4.

#### Capital cost expenses for photovoltaic farms

To assess the capital cost of a utility-scale solar system, a comprehensive capital cost calculator has been developed based on the U.S. solar PV system benchmarks reported by NREL and their corresponding “PV System Cost Model” (PVSCM) model.^28^

A 100-MW_DC_ utility-scale PV system with single-axis tracking, a central inverter with an inverter load ratio of 1.34 kW_DC_/kW_AC_, and crystalline silicon (c-Si) solar cell modules with an efficiency of 0.203 m^2^/kW_DC_ is used as the representative system for collecting values from the PVSCM model. Our capital cost calculator (see Figure S8) integrates these components and values, adjusting for the nameplate capacity of the system, to provide a detailed estimate of the total investment required for deploying a solar PV system of a given nameplate capacity (see Figure S9). The use of up-to-date benchmarks ensures that the calculations reflect current market conditions and technological advancements.

#### Capital cost expenses for battery energy storage

To estimate the capital cost variation of different LIB systems, we leverage the capabilities of the Battery Performance and Cost (BatPaC Version 5.1) tool, developed by Argonne National Laboratory.^29^ Using this tool, we estimate the capital costs (see Table S5) for building a battery system (see Table S4) using the leading three chemistries (for the LIBs we consider, we assume that the negative electrode material is always lithiated graphite. As such, here “chemistry” in this work refers to a choice of positive electrode material. Other choices could be made for the negative electrode, which could constitute a different “chemistry”.) currently used in the industry: NMC (811), NCA, and LFP.^30^

To obtain a capital cost estimate for the battery pack using a given chemistry, BatPaC requires the user to specify certain details of the battery pack. Here, we consider a battery pack with a duration of 4 h, obtained by appropriately specifying the target power and the rated energy of the pack. As utility-scale storage is a long-duration application, we consider only energy-type cells in our analysis.

The BatPaC analysis generally yields cost numbers that are significantly less than the average cost given in the literature, as measured by Mauler et al.^30^ To align with literature estimates and predictions while retaining the relative cost differences between chemistries, we take the cost of a pack rated at 1MW_DC_ power produced by BatPaC, average this cost over a range of rated durations, and find a scale factor to the average cost of that chemistry reported in the Mauler et al. report.^30^ This scale factor is then applied uniformly to all higher power ratings and durations for a given chemistry.

In addition to the cost of the battery storage unit, we also account for the costs associated with installation and integration of the battery storage unit (see Table S6) in obtaining the overall cost of the battery system (see Figure S10). These additional costs are further explained, and the total system cost is computed (see Figures S11 and S15) in supplemental information: Methods S1 – Section 4.

#### The total cost of ownership

To quantify the techno-economic benefits and costs of a microgrid deployment with solar and battery systems, the total cost of ownership (TCO) of the system is calculated as the sum of initial costs (ICs) and annual operating costs (AOCs) over the years of operation (YrsOp). As expected, the initial costs scale with the solar and battery system sizes (see Figure S11). The total electricity cost (TEC) is key for all stakeholders and depends on two metrics: the levelized cost of photovoltaic recharge (LCOPR) and the levelized cost of storage (LCOS).^31^ LCOPR reflects the cost of solar energy dispatch based on the capital cost and total energy output over the solar plant’s life. LCOS measures the battery system’s discharge cost, based on capital cost and the total energy discharged throughout the battery’s life, informed by the number of charge-discharge cycles that can be realized until the end of life. In addition, the TEC also reflects the average utilization of each resource (see Figures S13 and S14) to meet the excess load demand.

The LCOS value, averaged over a range of battery sizes between 10 and 100 MWh (see Figure S12), for different chemistry choices is shown in Table 2 under different optimistic, average, and pessimistic assumptions on the cycle lifetime of each chemistry (see Table S7). Due to its high expected lifetime, the LCOS of LFP is lower than the other chemistries despite its high capital cost. In contrast, NCA has the highest LCOS of all 3 chemistries due to its high capital cost and low expected lifetime.

**Table 2:** Levelized cost-of-storage averaged for each battery chemistry

Battery chemistry	LCOS_optimistic_ ($/MWh)	LCOS_average_ ($/MWh)	LCOS_pessimistic_ ($/MWh)
NMC	107.57	147.91	336.16
NCA	170.28	267.58	681.10
LFP	53.92	69.20	209.70

## Results and discussion

### Fleet operator cost metric

The fleet operator’s cost metric is the TCO per mile for a fleet of HDCVs that uses the alternative energy network. Here, the TCO is calculated for a fleet of 2,091 trucks. The initial cost for each vehicle in the fleet includes the Manufacturer’s Suggested Retail Price (MSRP) and the registration cost but is offset, slightly, by any existing government subsidies. At the end of the fleet’s assumed 5-year operational lifetime, vehicles retain a residual value based on a percentage of the MSRP. The fleet operator’s AOC includes insurance costs per vehicle, vehicle maintenance costs, and the cost of electricity used by the fleet. The TCO for the full fleet is calculated by summing these costs. Dividing this TCO by the total vehicle miles traveled by the fleet over the 5 years of expected operation yields the fleet operator’s cost metric. We additionally account for a penalty to account for the increased cost of dwell time to charge, as well as the payload capacity loss for electrified HDCVs, which are generally heavier due to the on-board battery.^32^^,^^33^ Further details on the computation of the fleet operator cost metric and assumptions (see Tables S8 and S9) may be found in supplemental information: Methods S1 – Section 4.

As a point of comparison, we evaluate the TCO/mile for a diesel-based HDCV fleet using the same framework above, substituting diesel in place of electricity as the fuel source. We find that the cost of operating a diesel-based fleet in the Port of Savannah region is ∼$1.20/mile. This serves as a benchmark against which the costs of an electrified fleet can be assessed.

### Capability and techno-economic analysis of viable microgrid deployment sites

In this section, we examine the potential impact of deploying microgrids, comprising solar and battery energy storage, at each of the 171 viable sites identified by our siting framework. Specifically, we assess how such deployments could help mitigate reliance on conventional fossil-fuel based energy sources. We then analyze the associated deployment costs and explore the tradeoffs faced by fleet operators.

#### Site-specific maximum air quality improvement potential

To perform the resource dispatch optimization for each viable site, we use the estimated hourly available solar capacity profile for that site. We note that while this analysis yields an estimate for the maximum potential for displacing conventional energy use at a given site, a lower level of displacement can always be achieved by developing a solar farm with a nameplate capacity below the site’s maximum potential. An extended sensitivity analysis on how the variation of solar and battery system size affects the capabilities (see Figures S18–S25) and deployment costs at a given site, is performed in supplemental information: Methods S1 – Section 6. Our sensitivity analysis reveals that the larger the solar nameplate capacity and battery system, the greater the expected gains in air quality, and thus justifies our focus on the maximum potential here. Thus, hereafter, we will consider two cases for each viable site: (1) a microgrid is deployed with a solar system alone, and (2) a microgrid is deployed with both solar and a 100 MWh battery energy storage system.

**Figure 5:**
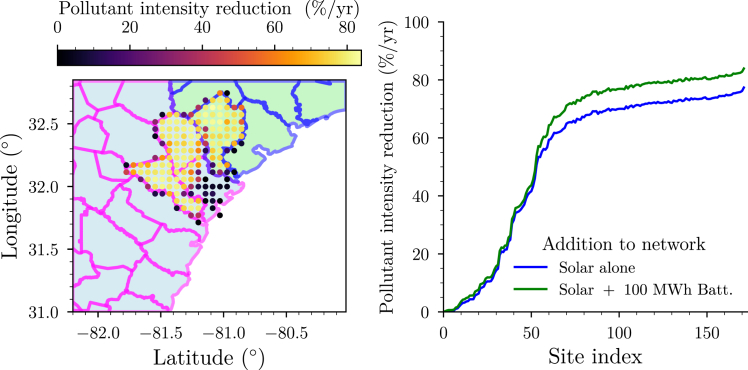
Pollutant intensity reduction potential of the viable sites in the Port of Savannah region (Left) Geographic locations of viable sites. Point colors: maximum total excess pollutant-intensity reduction, assuming the deployment of solar and a 100 MWh battery system. (Right) Maximum total excess air quality loss reduction for every site. Blue curve: only solar system deployed, no battery. Green curve: Solar system and 100 MWh battery deployed.

We find (Figure 5, right plot) that a maximum pollutant intensity reduction of approximately 77% is achievable by the largest nameplate capacity site with the addition of solar resources alone to the energy network. The addition of a 100 MWh battery at the same site further increases this reduction level to nearly 84%. Thus, at the site with the highest nameplate capacity, deploying a microgrid with solar and batteries could supply a significant portion of the electrified HDCV fleet’s load demand in the Port of Savannah region may be provided by alternative energy and energy storage resources, thereby significantly enhancing the air quality improvements associated with the electrified HDCV fleet.

#### Site-specific total cost of electricity

**Figure 6:**
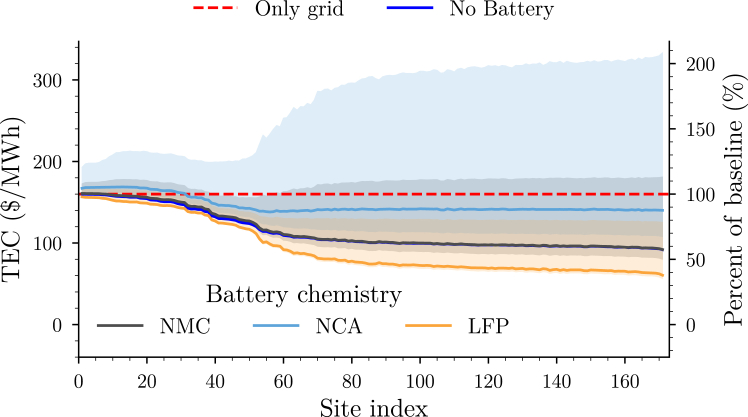
Total electricity cost (TEC) for viable solar sites in the Port of Savannah region Only grid (red dashed line): TEC of baseline network where only grid power is used to meet excess load demand. No battery (blue solid curve): microgrid deployed with only a solar system. Other colors denote different choices of chemistry when a 100 MWh battery is deployed along with the solar system. Shaded regions denote the minimum and maximum TEC uncertainty corresponding to the different LCOS scenarios listed in Table 2.

We estimate the TEC for all viable solar sites in the Port of Savannah region in Figure 6. The details of the overnight capital costs, site-specific LCOPR, LCOS, and average resource utilization that result in this TEC estimate may be found in supplemental information: Methods S1 – Section 5. The TEC of the baseline network (i.e., grid-only supply) is equivalent to the grid electricity price (GEP) of 160 $/MWh. As the LCOPR of ∼$26/MWh is significantly lower than the GEP in this region (Figure S16), we find that deploying solar alone generally improves the TEC over the baseline network. As sites with increasing nameplate solar capacity are considered, the TEC decreases further. This originates from a lower utilization of the grid resources in order to minimize unrealized air quality improvements.

Deploying a microgrid with an additional 100 MWh battery can increase or decrease the TEC relative to the case of the baseline network, depending on the choice of battery chemistry. We find that choosing LFP as the battery chemistry, across all the viable solar sites, results in a TEC lower than that of a microgrid with solar alone. This is due to the LCOS of LFP (Figure S12) being lower than the GEP: Using an LFP battery to provide power to the load is considerably cheaper than using the grid. In contrast, deploying an NMC battery increases the cost relative to just deploying solar alone. Although NMC has an LCOS marginally below the GEP, the additional system cost associated with both charging and discharging leads to a (small) net increase in TEC relative to solar alone. Due to its high LCOS relative to the GEP, the TEC of an NCA battery system deployed alongside a solar farm tends to be higher than that of the other chemistries. Overall, we find that LFP is the best choice of battery chemistry for this application in this region. Importantly, this result is highly sensitive to the assumption on battery system lifetime as can be observed by the wide variation in the expected TEC (shaded regions in Figure 6).

#### Site-specific fleet operator cost metric

**Figure 7:**
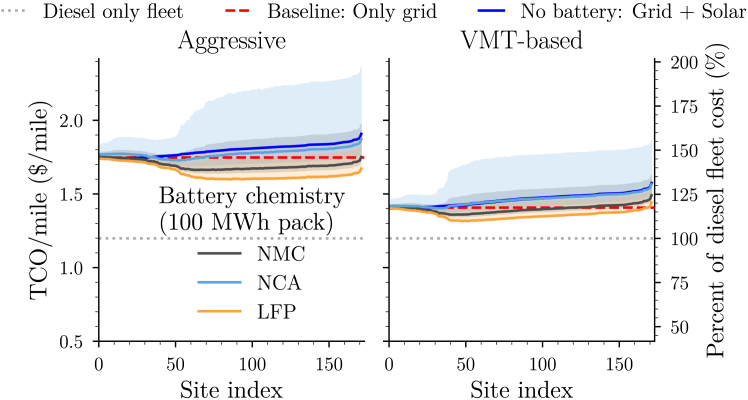
Cost per mile to fleet operator in the aggressive electrification scenario (left plot) and in the VMT-based scenario (right plot) Only grid (red dashed line): TEC of baseline network where only grid power is used to meet excess load demand. No battery (blue solid curve): microgrid deployed with only a solar system. Other colors denote different choices of chemistry when a 100 MWh battery is deployed along with the solar system. Shaded regions denote the corresponding uncertainty from the TEC seen in Figure 6.

We estimate the fleet operator TCO/mile for every viable microgrid deployment site in the Port of Savannah region. We first consider the *aggressive electrification scenario*: The fleet transitions the maximum number of trucks to electrified powertrains while maintaining coverage of all current routes in the region. Importantly, to ensure that this constraint is met, some diesel vehicles are needed in the fleet to meet the longer routes that electrified vehicles cannot attain. In this scenario, 1,631 are battery electric trucks and 460 are diesel trucks. We observe (Figure 7, left plot) that without microgrid deployment, the TCO/mile to the operator is approximately $1.75/mile. Therefore, electrification amounts to a ∼46% increase in cost per mile for the fleet operator. We find that the increase in cost can be mitigated with the deployment of a microgrid. In the best-case scenario, deploying at the largest nameplate capacity site and using an LFP battery storage solution, the cost can be lowered to ∼$1.60/mile, or equivalently, a ∼33% increase in price relative to a pure diesel-based fleet.

As the site index increases, the total energy cost (TEC) decreases due to the larger nameplate capacity of the microgrid, which lowers overall electricity costs (recall that increasing site index corresponds to increasing nameplate capacity). However, as the microgrid size continues to grow, the associated maintenance costs––which scales with the capital cost––eventually outweigh the savings from cheaper electricity. As these maintenance costs are passed on directly to the fleet operator, it becomes disadvantageous for the fleet to adopt an oversized microgrid to support its operations. This creates a practical “sweet spot” that balances this tradeoff.

As the aggressive electrification scenario results in significant cost increases to the fleet operator, a more conservative scenario sees fleet operators electrifying an even smaller portion of their fleets. Specifically, we consider a vehicle miles traveled (*VMT)-based scenario*: only trucks that attain VMTs of 60,000 miles or greater are electrified. This scenario results in only 801 trucks being electrified, with the others remaining as diesel trucks. The average annual VMT for the electrified trucks becomes 82,222 miles. Performing the analysis for this conservative scenario sees the TCO/mile lower significantly (Figure 7, right plot) to $1.32/mile, with the cost increase amounting to a ∼17% increase in TCO/mile relative to a fully diesel fleet in the no microgrid case and sees further reduction to $1.29/mile (or equivalently an 8% increase) building a microgrid at the lowest-cost site with an LFP battery. A further analysis on the costs to the utility (see Figure S26) over the lifetime of the fleet is found in supplemental information: Methods S1 – Section 7.

### Conclusion

The electrification of drayage heavy-duty commercial vehicles (HDCVs) at intermodal hubs such as ports is crucial for maximizing air quality improvements; however, this transition increases electricity demand and challenges the existing grid infrastructure. Without the deployment and integration of alternative energy sources, electrification may result in limited improvements in air quality.

We present an integrated framework that combines ORNL’s OR-SAGE siting tool and NREL’s reV renewable energy model to systematically identify viable sites for microgrid deployment and quantify each site’s energy potential. This framework incorporates an optimization component to maximize the air quality improvements achievable through microgrid deployment at a given site, along with a cost analysis module that evaluates how such microgrid deployments affect the total cost of ownership (TCO) for key stakeholders, particularly HDCV fleet operators.

We applied this framework to the region surrounding the Port of Savannah, Georgia (USA), identifying and assessing 171 viable sites for microgrid deployment. Our analysis shows that targeting sites with intermediate nameplate solar capacity yields the greatest expected air quality improvements at the lowest fleet lifetime cost to HDCV fleet operators. These findings highlight the framework’s utility in supporting decision-making by integrating geospatial siting, air quality optimization, and techno-economic analysis, thereby offering policymakers, utilities, and fleet operators a systematic method for identifying cost-effective, pollutant intensity-reducing microgrid deployment opportunities.

The conclusions drawn here are contingent on several underlying assumptions, including estimates of alternative energy deployment costs, site-specific siting constraints, alternative resource availability, and assumed microgrid system lifetimes. While these assumptions introduce uncertainties, the modular structure of the framework enables sensitivity analyses and the exploration of alternative cost and technology scenarios, making it adaptable for broader regional or national-scale applications.

### Limitations of the study

While our framework demonstrates the capability to assess the impact and cost of microgrid deployment in a single region, several limitations remain that suggest fruitful directions for future research. First, our analysis considers only a single microgrid deployment; extending this to multiple coordinated microgrids, deployed either front-of-the-meter or behind-the-meter, could reveal network-level effects on both cost and air quality outcomes, particularly from the fleet operator’s perspective. Second, although we focus on solar photovoltaics and lithium-ion batteries, diversifying the resource mix to include wind, hydro, nuclear, or emerging long-duration energy storage technologies (e.g., flow batteries or thermal storage) could significantly alter system performance and economic outcomes, especially in regions where solar is less viable. Third, our dispatch optimization assumes perfect foresight of solar availability and vehicle charging demand. Incorporating uncertainty, forecast errors, and real-time responsiveness would make the analysis more realistic and could impact both operational strategy and economic viability. Fourth, we model battery systems using fixed cycle lifetimes, omitting degradation effects. This simplification may understate performance losses, replacement costs, and life cycle costs. Finally, we do not model integration with vehicle charging networks or explore vehicle-to-grid (V2G) capabilities. Microgrids that interact with fleets may benefit from using vehicle batteries as distributed energy storage, managed through appropriate state-of-charge constraints and dispatch control permissions.

Our results around the Port of Savannah show that microgrids can address grid limitations while also improving air quality. However, the regional heterogeneity of grid pollution intensity and costs means the benefits of microgrid deployment will vary across the U.S. A national-scale extension of this framework could identify regions where microgrids are most cost-effective and environmentally impactful, aiding policymakers and industry stakeholders in prioritizing deployments that maximize air quality improvements in the freight transportation sector.

## Resource availability

### Lead contact

Further information and resource requests should be directed to the lead contact, Vivek A. Sujan (sujanva@ornl.gov).

### Materials availability

This study did not generate new materials.

### Data and code availability

• Data: All data used to generate the figures in this work are available at Zenodo: https://doi.org/10.5281/zenodo.16423277.
• Code: Custom analysis scripts are available at Zenodo: https://doi.org/10.5281/zenodo.16423277.
• Other items: The OR-SAGE tool uses proprietary data sources; The reV model is entirely public, and we have reported the technological parameters we use in the supplemental information (Section 3); Battery unit costs are estimated using BatPaC v5.1, with assumptions found in supplemental information: Methods S1 – Section 5. The tool is available from Argonne National Laboratory at: https://www.anl.gov/partnerships/batpac-battery-manufacturing-cost-estimation; The TCO calculator for the fleet costs incorporates protected information and thus cannot be released publicly. Access may be obtained by contacting the lead contact, Vivek A. Sujan (sujanva@ornl.gov).

## Acknowledgments

This article has been authored by UT-Battelle, LLC under Contract No. DE-AC05-00OR22725 with the U.S. Department of Energy. The United States Government retains, and the publisher, by accepting the article for publication, acknowledges that the United States Government retains a non-exclusive, paid-up, irrevocable, worldwide license to publish or reproduce the published form of this article, or allow others to do so, for United States Government purposes. DOE will provide public access to these results of federally sponsored research in accordance with the DOE Public Access Plan (https://www.energy.gov/doe-public-access-plan). This research was supported by the 10.13039/100000015U.S. Department of Energy (DOE) Vehicle Technologies Office under award WBS 7.2.0.502/FWP CEVT442, administered by Oak Ridge National Laboratory's (ORNL) National Transportation Research Center. Additional support was provided through an appointment to the ORNL Graduate Research Opportunities (GRO) Program, sponsored by DOE and administered by the Oak Ridge Institute for Science and Education. The DOE technical management team included Patrick Walsh, Raphael Isaac, Laura Robertson, and Casey Roepke. This research used resources of the Compute and Data Environment for Science (CADES) at the Oak Ridge National Laboratory, which is supported by the Office of Science of the U.S. Department of Energy under Contract No. DE-AC05-00OR22725. Some of the computing for this project was performed on the Sherlock cluster. We would like to thank 10.13039/100005492Stanford University and the Stanford Research Computing Center for providing computational resources and support that contributed to these research results.

## Author contributions

Conceptualization, J.N.E.L. and V.A.S.; methodology, J.N.E.L., R.S., B.A.M., and V.A.S.; data curation, J.N.E.L. and B.A.M.; software, J.N.E.L., B.A.M., and V.A.S.; visualization, J.N.E.L., B.A.M., and S. O.; writing – original draft, J.N.E.L.; writing – review and editing, J.N.E.L., R.S., B.A.M., S.O., and V.A.S.; supervision, S.O. and V.A.S.; project administration and funding acquisition, S.O. and V.A.S.

## Declaration of interests

The authors declare no competing interests.

## STAR★Methods

### Key resources table

**Table:** 

REAGENT or RESOURCE	SOURCE	IDENTIFIER
**Deposited data**		

cb_2018_us_county_500k.shp	US Census Bureau	https://www.census.gov/geographies/mapping-files/time-series/geo/carto-boundary-file.html
gac_sol_capacity_profiles.csv gac_sol_supply-curve-aggregation.csv	National Solar Radiation Database, reV model, Authors	https://nsrdb.nrel.gov/ https://nrel.github.io/reV/
SolarCapEx.xlsx	PV System Cost Model (PVSCM) Authors	https://www.energy.gov/eere/solar/solar-photovoltaic-system-cost-benchmarks
BatteryStorageCapEx.xlsx	BatPaC Authors	https://www.anl.gov/partnerships/batpac-battery-manufacturing-cost-estimation
processed_results_siteVariation.npz processed_results_siteVariation2.npz processed_results_hypothetical.npz	Authors	https://github.com/jnlucero96/DER_augmentation/tree/main

### Method details

#### Quantifying fleet electrification impacts

We used the OR-AGENT framework to model fleet electrification scenarios for heavy-duty commercial vehicles (HDCVs) operating in the Port of Savannah region. OR-AGENT generates hourly estimates of energy demand based on travel patterns, fleet size, and battery capacity assumptions. To understand the implications of electrification, we calculated unrealized air quality improvements by overlaying these additional electricity demands onto county-level grid load and pollutant intensity profiles. Expanded descriptions of the workflow and representative hourly pollutant intensity profiles are provided in Figures S1–S3 and Methods S1.

#### Grid–solar–battery integration framework

To mitigate the unrealized air quality improvements associated with grid reliance, we developed a dispatch model that integrates grid supply with utility-scale solar photovoltaic (PV) generation and battery energy storage. The model was formulated as a mixed-integer linear program (MILP) that optimizes hourly energy flows to maximize air quality improvements while ensuring that excess fleet demand is always met. Solar energy could be routed directly to the fleet load, stored in batteries, or curtailed if generation exceeded system capacity. Batteries could be charged by either solar or grid power and were discharged to offset peak demand. Lithium-ion battery efficiency was assumed to be 85% roundtrip. This framework captures the interaction between variable alternative energy resources and fixed fleet demand, highlighting when storage provides additional value. Mathematical formulations, optimization parameters, and representative dispatch profiles are provided in Table S1*,* and Methods S1.

#### Siting framework for photovoltaics

The feasibility of solar deployment was assessed using a siting workflow that combines the Oak Ridge Siting Analysis for Generation Expansion (OR-SAGE) with the National Renewable Energy Laboratory’s Renewable Energy Potential (reV) model. We first applied geospatial exclusion criteria to identify land areas that are unsuitable for solar deployment, including wetlands, floodplains, slopes steeper than 5%, protected lands, and densely populated regions. The remaining land was classified as “viable” for PV siting. Using the reV model, we then estimated hourly solar generation potential for each viable site based on the National Solar Radiation Database (NSRDB 2019). This dual-model approach generates both a spatial map of candidate sites and a temporal profile of their expected hourly power output. Expanded siting maps, site-level capacity distributions, and technical explanations are provided in Tables S2–S3*,* Figures S4–S7*,* and Methods S1.

#### Cost estimation framework

We develop a cost estimation framework to assess the financial investment required to integrate solar and battery systems into the regional grid for drayage fleet electrification. Solar capital costs were calculated as the sum of equipment (modules, inverters, balance-of-system components) and development expenses (installation, labor, permitting, and project management). For batteries, costs were estimated using Argonne’s BatPaC tool for three lithium-ion chemistries: NMC 811, NCA, and LFP, which were chosen to capture trade-offs in capital cost and cycle life. Additional integration costs were included for power conversion, controls, construction, and grid interconnection. To enable comparison across technologies, we applied a total cost of ownership (TCO) framework, normalizing TCO by miles traveled to represent the cost borne by fleet operators. Complete parameter ranges, assumptions, and chemistry-specific cost breakdowns are provided in Tables S4–S9, Figures S8–S12, and Methods S1.

#### Usage patterns and sensitivity analysis of microgrid size assumptions

In the Port of Savannah region, 171 identified sites were evaluated for their potential to reduce excess air quality losses through fleet electrification under two scenarios: solar-only and solar paired with 100 MWh of collocated battery storage. Results are based on an optimal dispatch strategy where solar is prioritized to offset grid dependence, though reductions plateau at roughly 50%. To achieve further air quality loss reductions, batteries were found to be most effective when charged by solar more than 80% of the time; however, limited solar availability at smaller sites necessitated some reliance on grid charging. The levelized cost of photovoltaic recharge (LCOPR) across all sites averaged $26.40/MWh. A sensitivity analysis was also conducted to explore the role of microgrids and to test sizing assumptions for both solar and batteries. For a representative site adjacent to the Port of Savannah, results show that larger solar capacities combined with larger batteries yield the greatest reductions in excess air quality losses. Detailed outcomes of these sizing studies are provided in Figures S13–S25 and Methods S1.

### Quantification and statistical analysis

Optimizations were performed using MATLAB R2025a. All analyses were performed using Python (v3.12.2) with standard scientific computing libraries. Statistical analyses consisted of scenario-based comparisons of air quality loss reduction, total cost of ownership, and electricity costs. Details of input datasets, computational methods, and software are provided in the supplemental information (Methods S1: Sections 3–4). This study did not use inferential statistics; all results derive from deterministic optimization and simulation analyses.
