# Supplementary material for: A microgrid deployment framework to support drayage electrification
Source: iScience. 2025 Oct 16;28(11):113804. doi: 10.1016/j.isci.2025.113804 (PMC12651681; doi:10.1016/j.isci.2025.113804)
Supplement: Document S1. Figures S1–S26, Tables S1–S9, and Methods S1 [file mmc1.pdf]

iScience, Volume *28*

Supplemental information

A microgrid deployment framework

to support drayage electrification

Joseph N.E. Lucero, Ruixiao Sun, Brandon A. Miller, Simona Onori, and Vivek A. Sujan

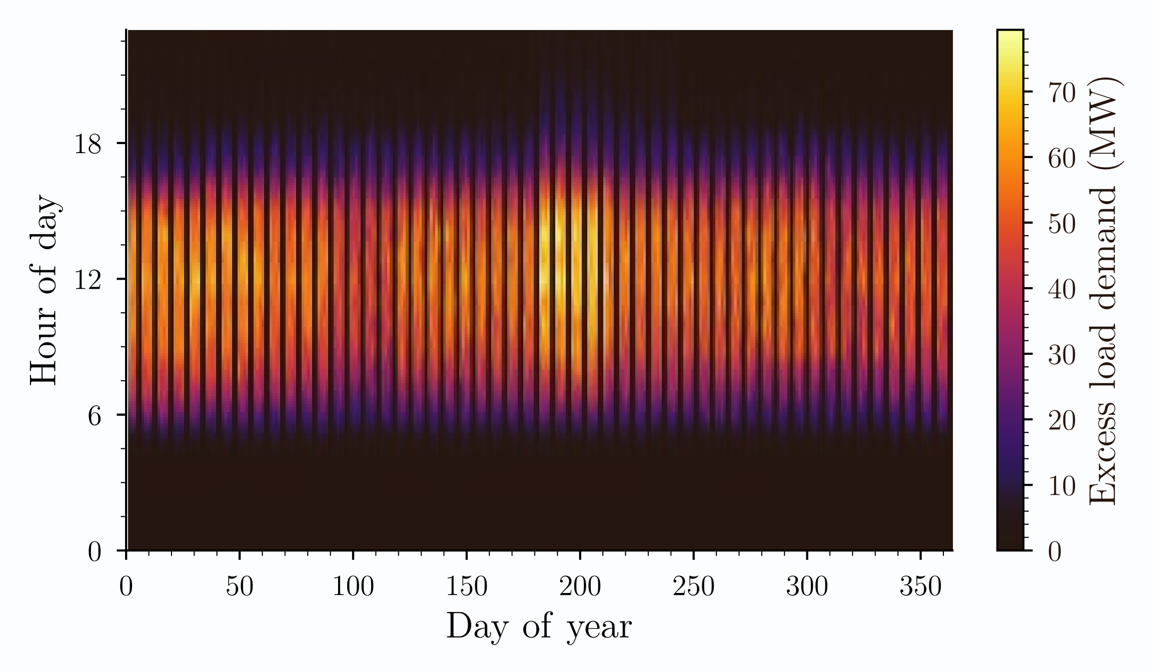

Figure S1: ***Excess load demand from a fleet of electrified HDCVs operating in the region around Port of Savannah, Related to Figure 2 in the main text.***

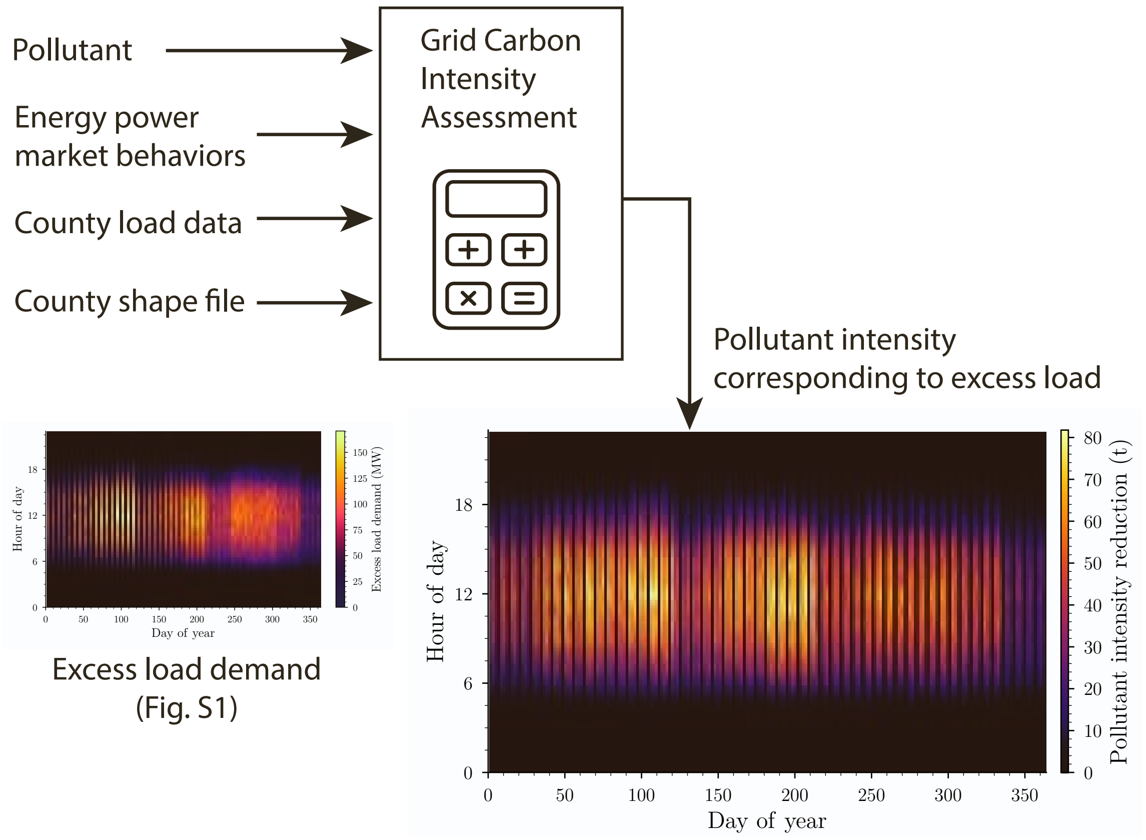

Figure S2: ***Schematic of pollution intensity estimation pipeline, Related to Figure 1 in the main text.***

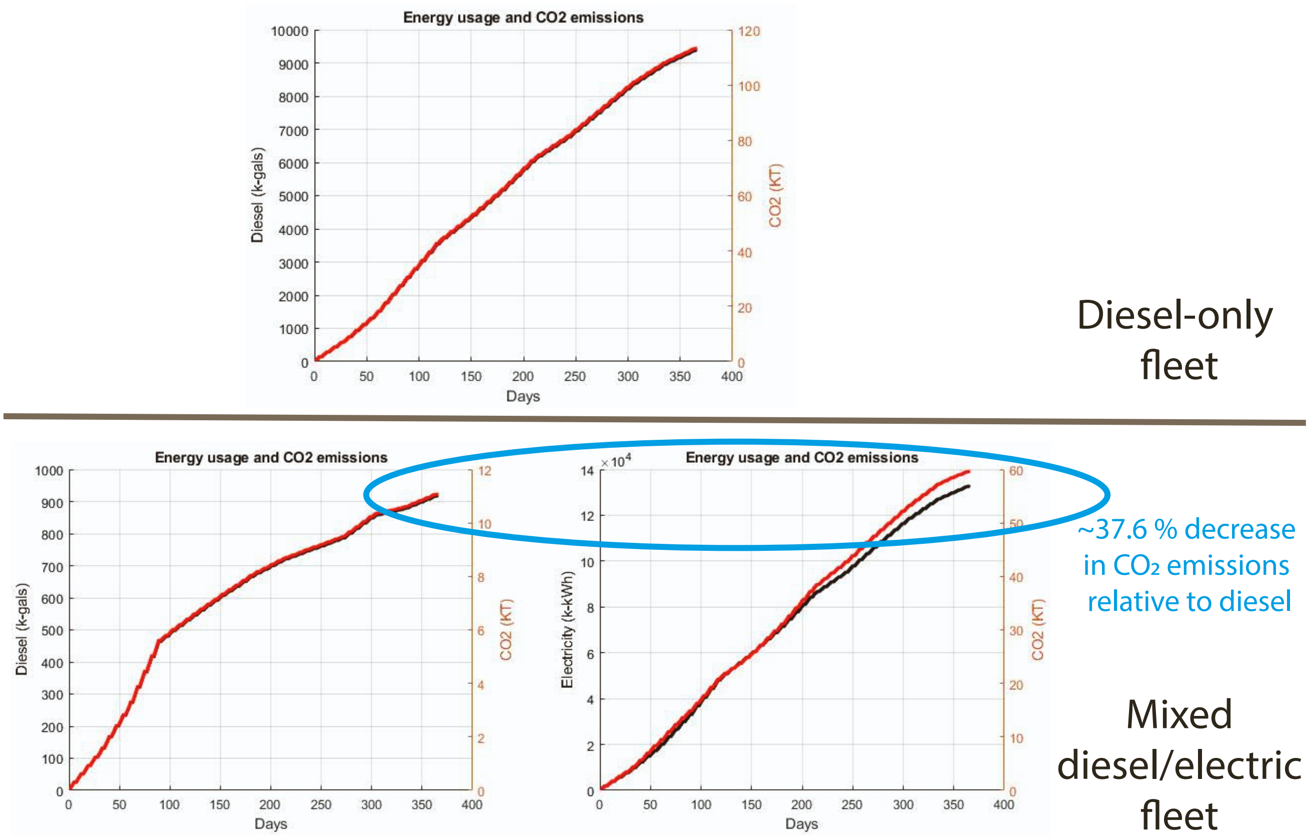

Figure S3: ***Energy usage and net pollution generated for diesel-based and mixed HDCV fleet operating in the Port of Savannah region, Related to Figure 1 in the main text.*** *(Top plot) Fully diesel-based HDCV fleet. (Bottom plots) Mixed HDCV fleet. Bottom left plot: energy usage and pollutant generation of the diesel vehicles in the mixed fleet. Bottom right plot: energy usage and pollutant generation for the BEV vehicles in mixed fleet. Each plot: left axis (black) is the fuel/electricity consumption, and right axis (red) is pollutant generated.*

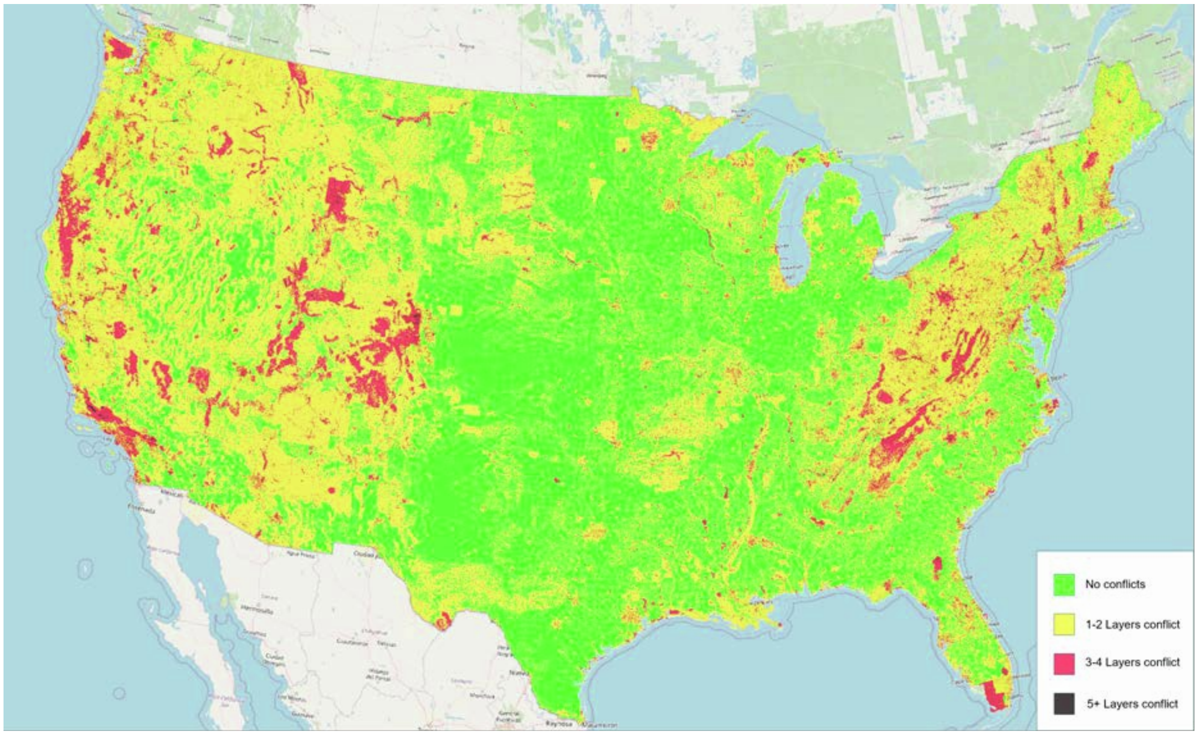

Figure S4: ***Composite siting map depicting the number of conflicting decision layers for the conterminous US, Related to Figure 3 in the main text.***

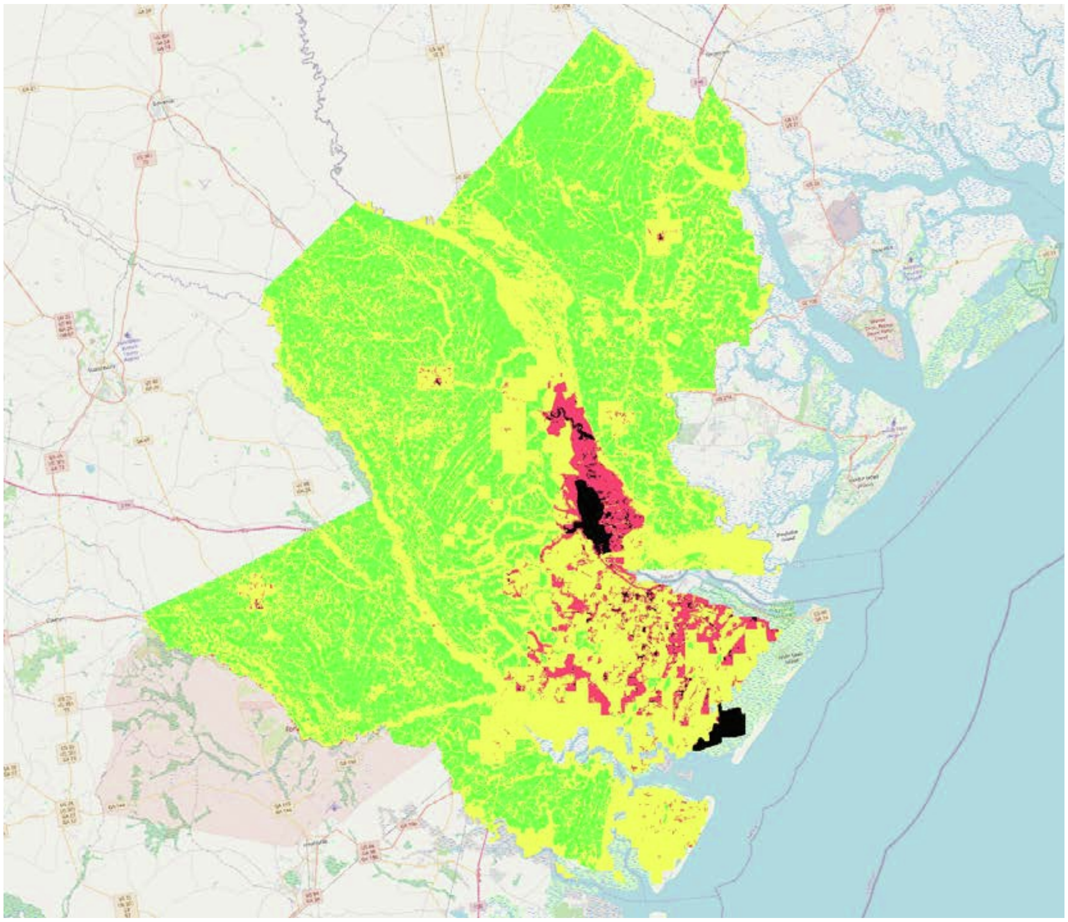

Figure S5: ***Composite map of siting conflicts for Chatham County, Georgia, and surrounding counties, Related to Figure 3 in the main text.*** *Green denotes regions viable for microgrid siting. Yellow, Red and Black indicate regions that have conflicts with land-use restrictions and thus are considered not viable for siting*

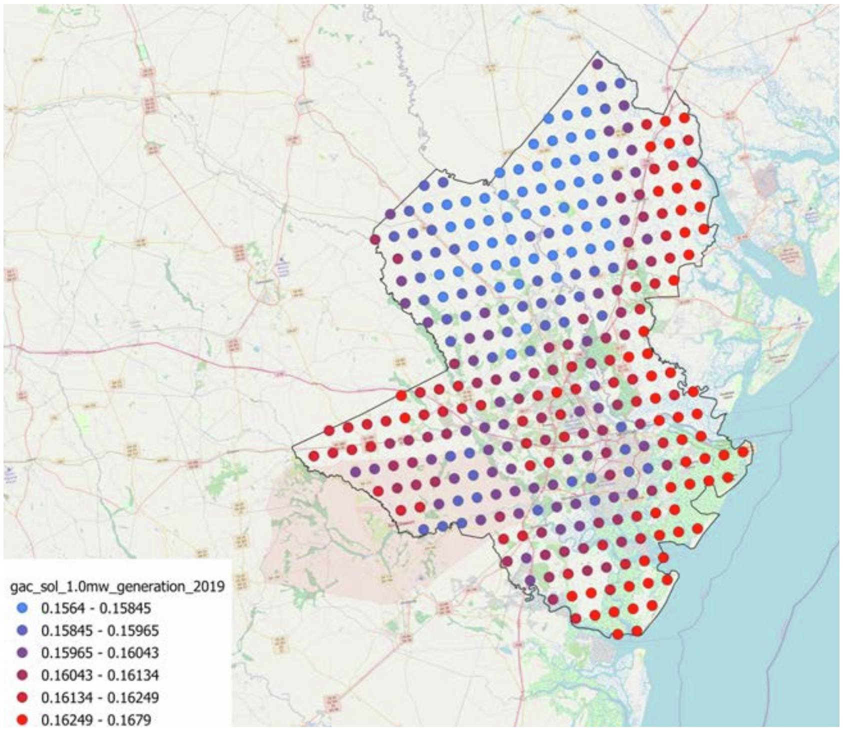

Figure S6: ***Capacity factors for Chatham County and the surrounding counties, Related to Figure 3 and Figure 4 in the main text.***

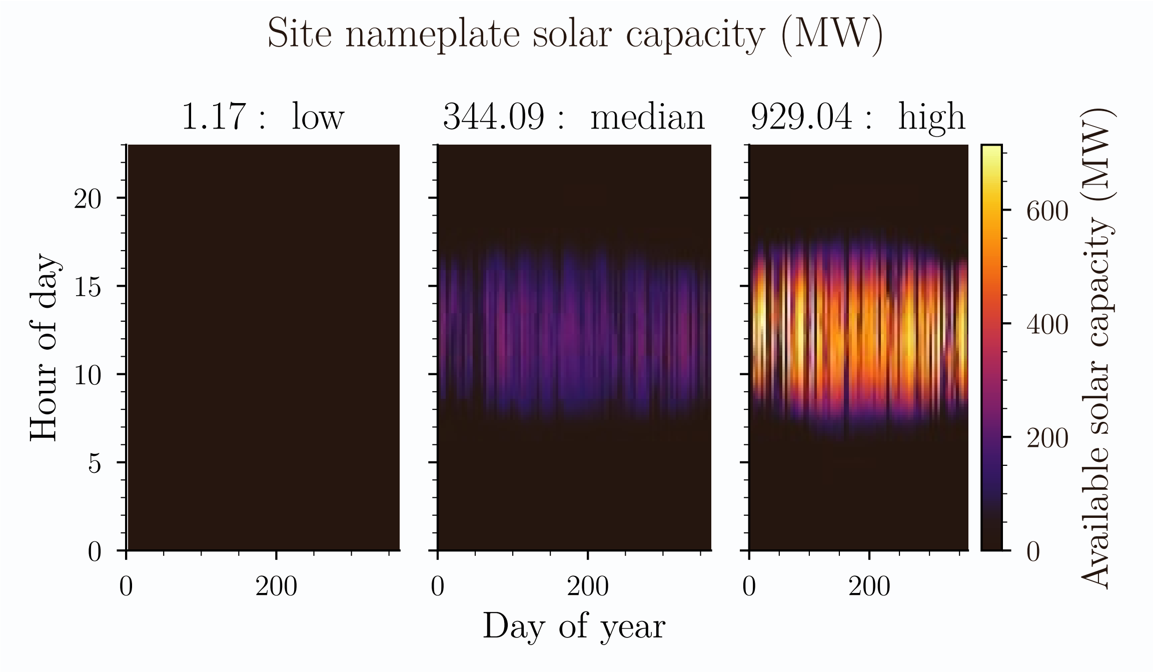

Figure S7: ***Estimated hourly available solar capacity generated from the proposed framework for three different representative sites in the Port of Savannah area, Related to Figure 4 in the main text.***

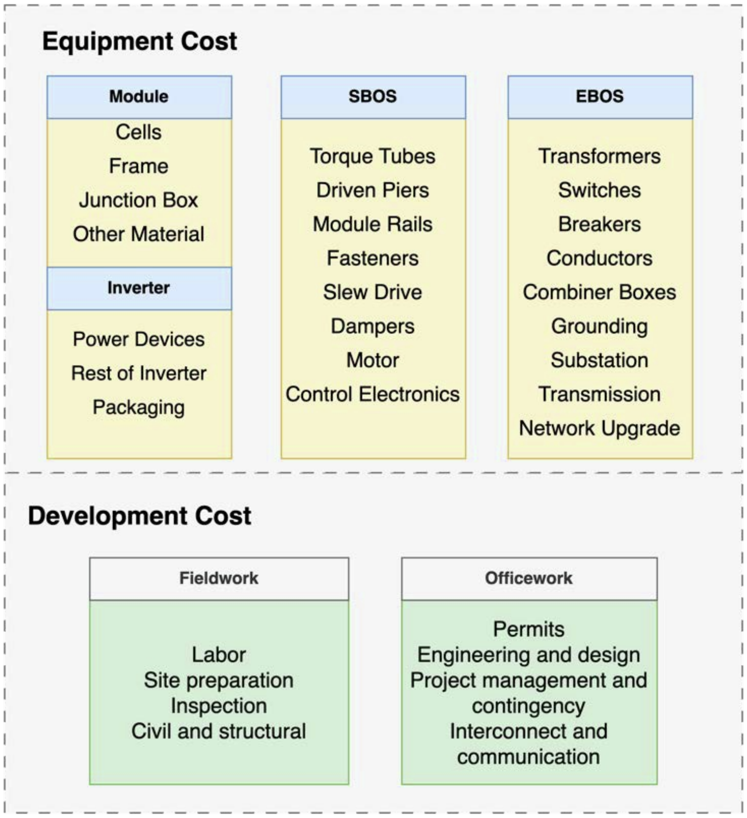

Figure S8: ***Primary components of solar installation cost. Related to Figure 6 and Figure 7 in the main text.***

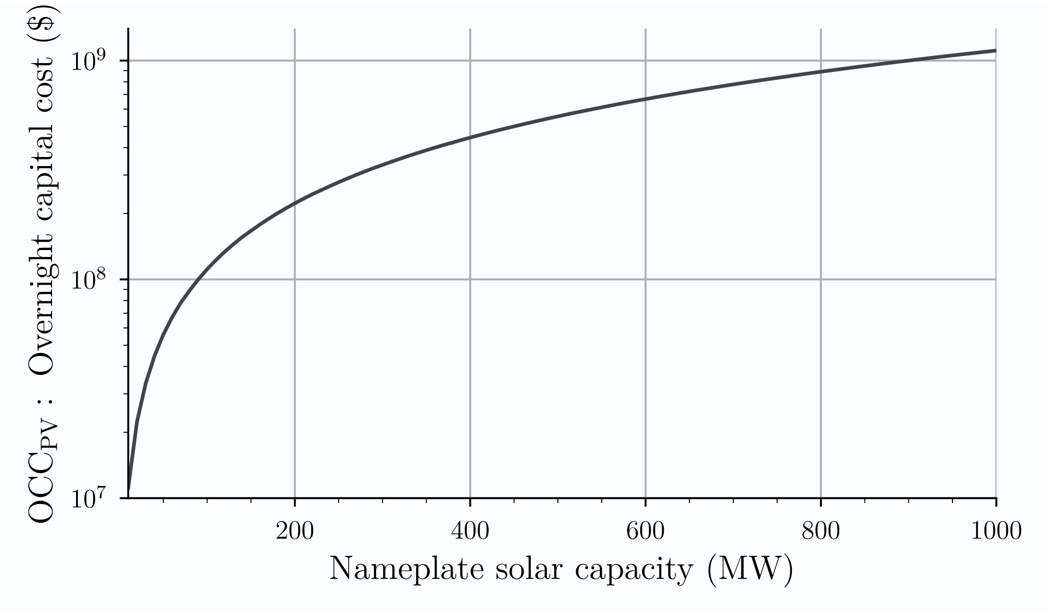

Figure S9: ***Capital cost associated with deploying a solar farm of a given nameplate solar capacity, Related to Figure 6 and Figure 7 in the main text.***

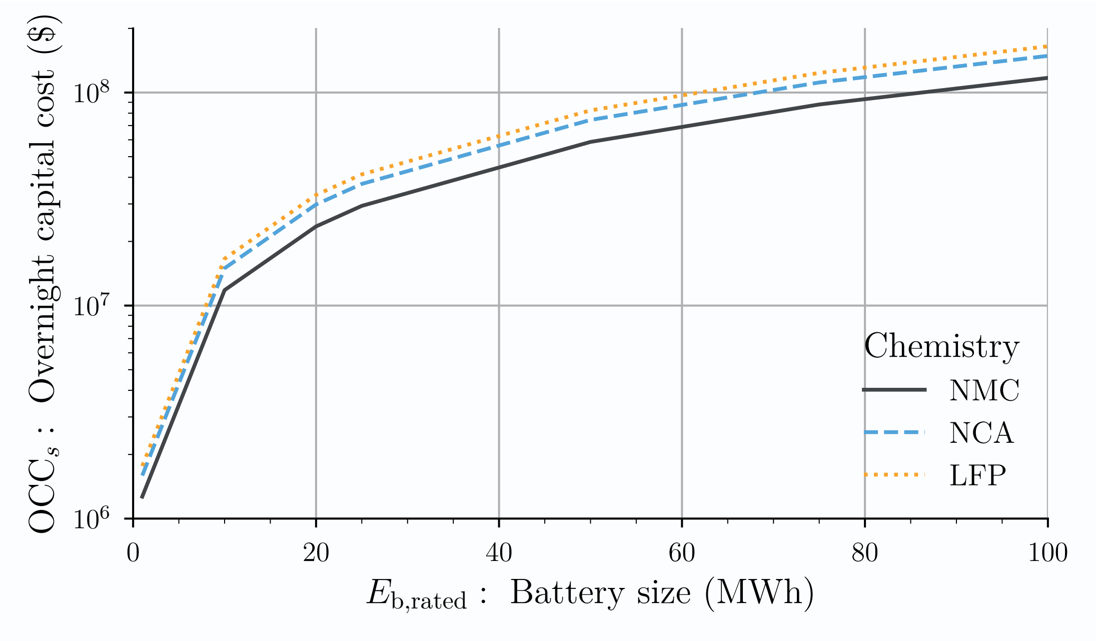

Figure S10: ***Cost of deployment for battery systems of different sizes for different chemistries, Related to Figure 6 and Figure 7 of the main text.***

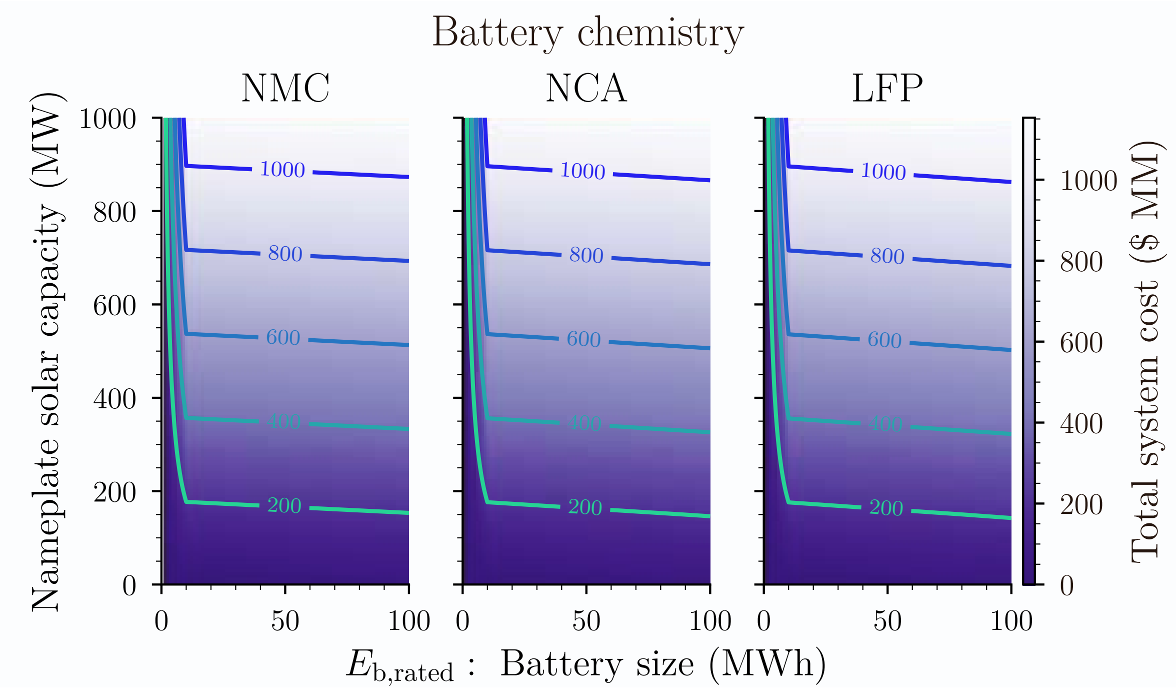

Figure S11: ***Total overnight capital cost (in millions USD) of deploying solar with a given nameplate solar capacity and an attached battery system of a given size, Related to Figure 7 in the main text.*** *Contour lines show constant-cost levels across the surface.*

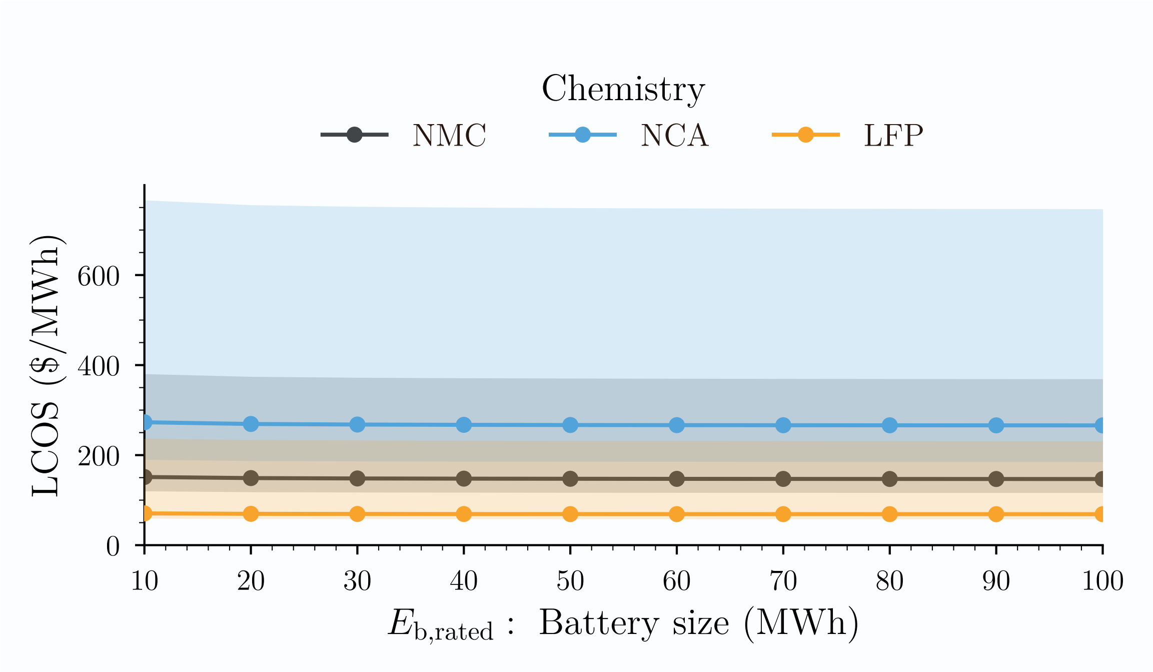

Figure S12: ***Levelized cost of storage (LCOS) for different size battery systems, Related to Figure 6 and Figure 7 of the main text.*** *Different colors correspond to different battery chemistries. Shaded region bounds the best and worst-case LCOS for each chemistry and size.*

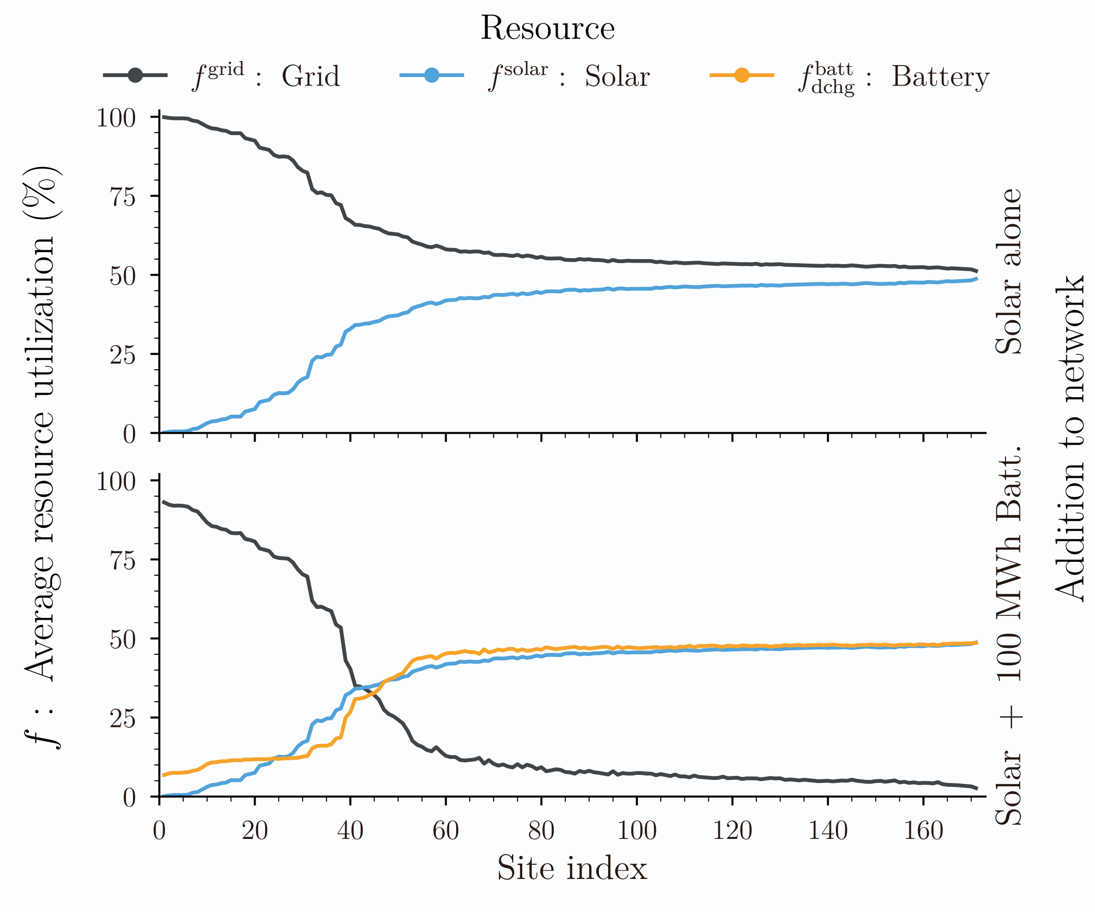

Figure S13: ***Breakdown of resource usage for each site in the region to power the excess load demand, Related to Figure 6 and Figure 7 of the main text.*** *(Top plot) Only solar installation deployed at site. (Bottom plot) Solar installation and 100 MWh battery deployed at site.*

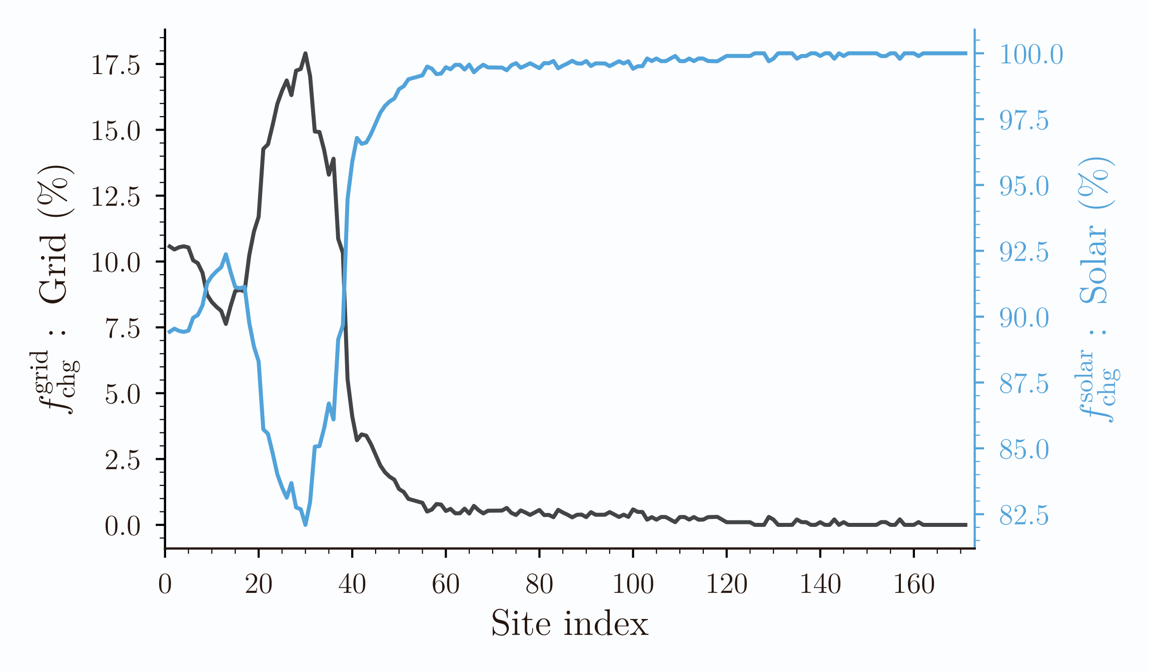

Figure S14: ***Average resource utilization to charge a 100 MWh battery for each viable site in the region.*** *(Left axis, black curve) Grid average utilization. (Right axis, blue curve) Solar average utilization, Related to Figure 6 and Figure 7 of the main text.*

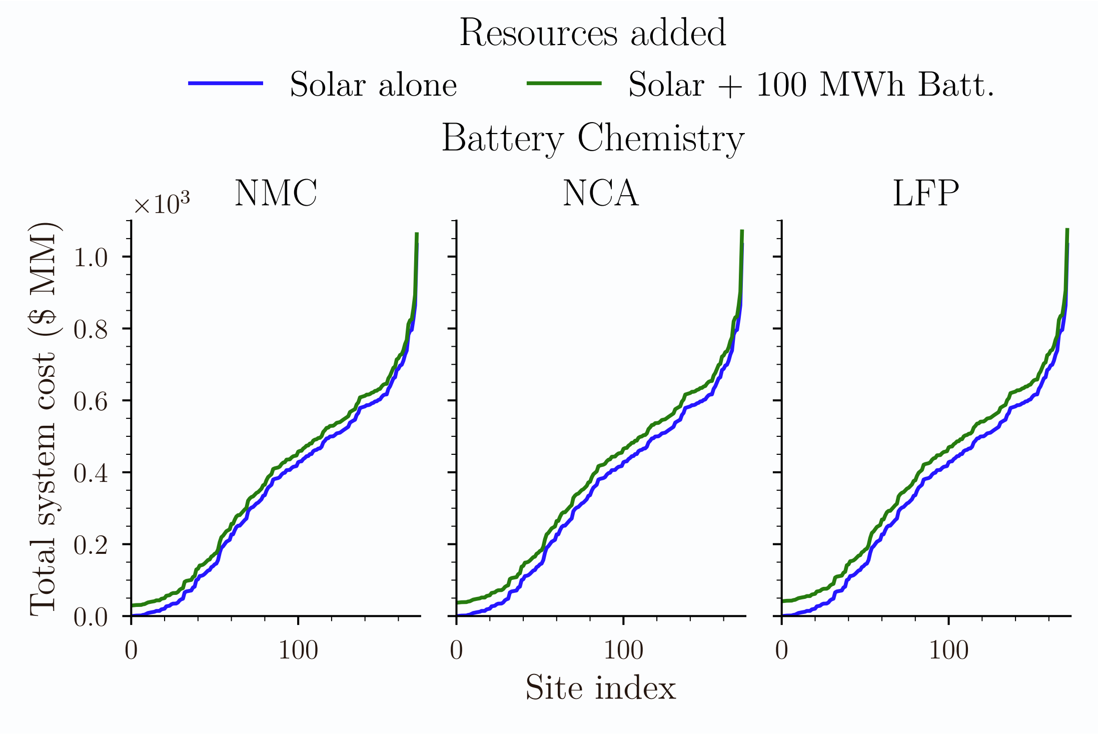

Figure S15: ***Total system cost of deploying a microgrid with solar farm alone (blue curve) and a microgrid with a solar farm and a 100 MWh battery system (green curve) for each of the viable sites within the region, Related to Figure 6 and Figure 7 of the main text.***

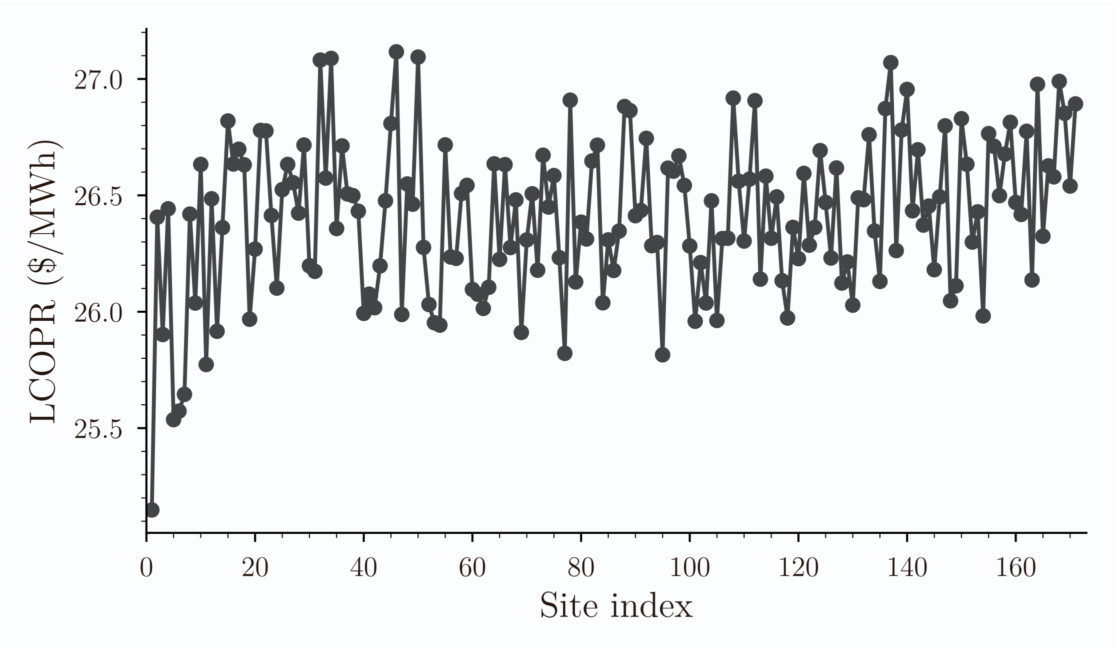

Figure S16: ***Levelized cost of photovoltaic recharge (LCOPR) for each of the viable sites in the region, Related to Figure 6 and Figure 7 of the main text.***

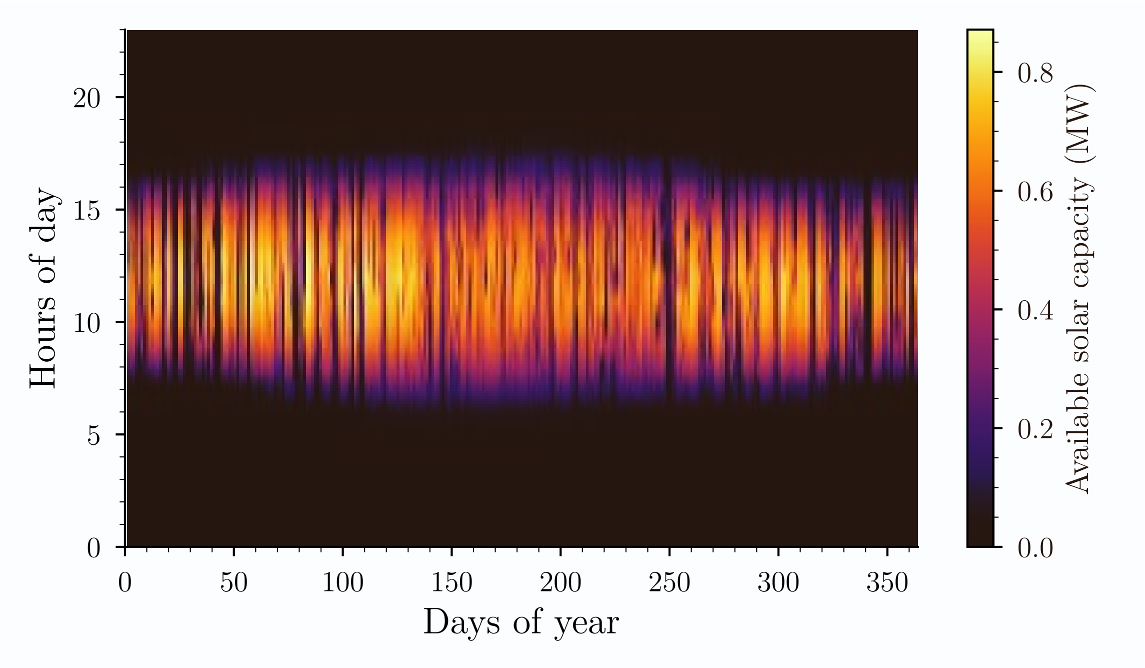

Figure S17: ***Hourly available solar capacity for a hypothetical 1 MW nameplate solar capacity site, deployed adjacent to the Port of Savannah, Related to Figure 4 of the main text.***

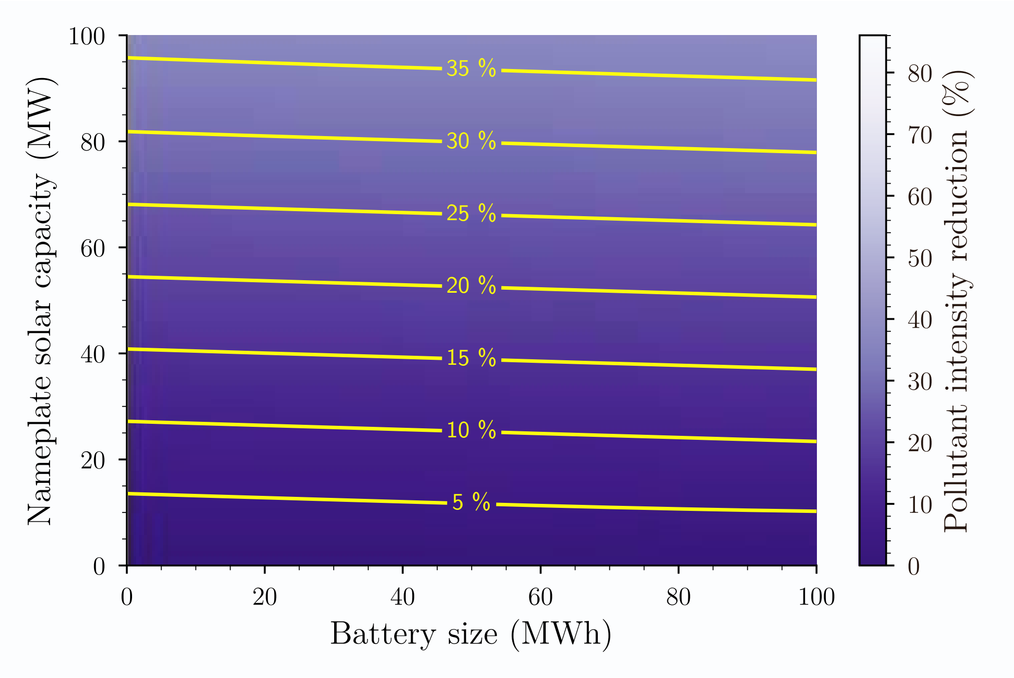

Figure S18: ***Maximum excess pollution reduction for a solar farm & battery system built just outside of the Port of Savannah, Related to Figure 5 in the main text.*** *Contour lines represent isolines of constant excess pollution reduction cost over the plotted surface.*

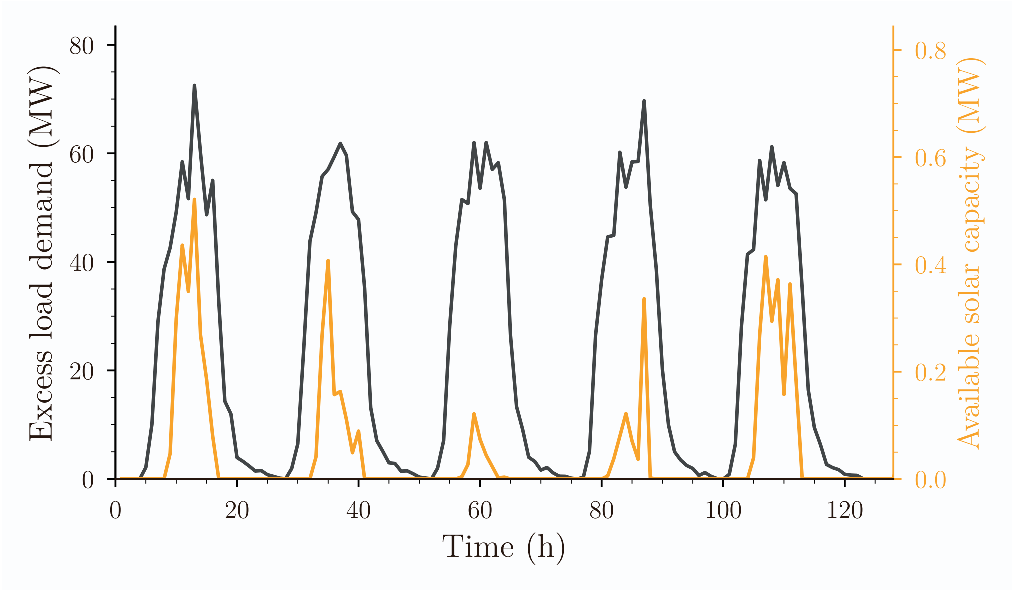

Figure S19: ***The peak of solar availability at the hypothetical site is generally aligned with the peak of the excess load demand, Related to Figure 5 in the main text.***

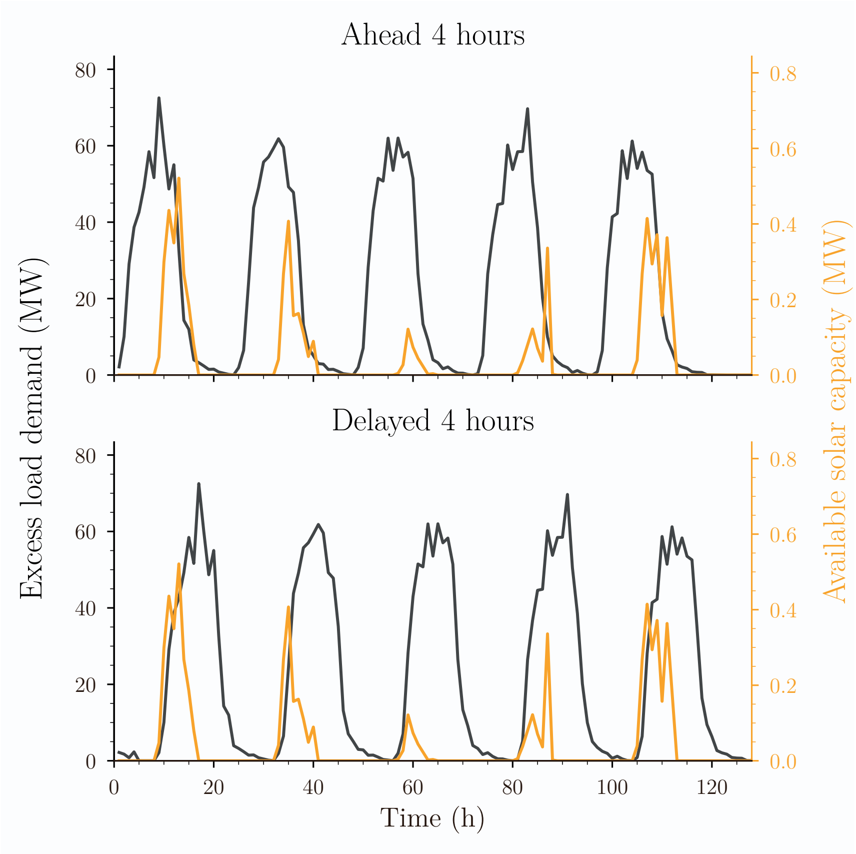

Figure S20: ***Artificially shifting the peak of excess load demand with respect to the peak of solar availability, Related to Figure 5 in the main text.*** *(Top plot) Excess load demand is shifted 4 hours ahead of the peak of solar availability. (Bottom plot) Excess load demand is delayed 4 hours relative to the peak of solar availability.*

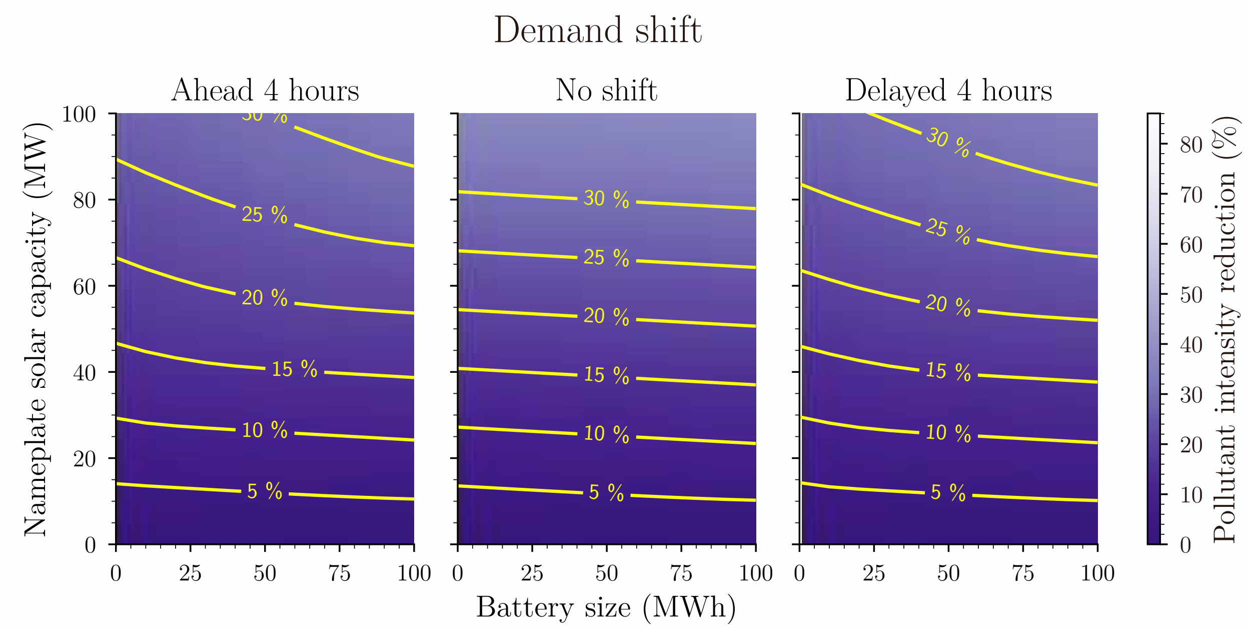

Figure S21: ***Maximum total excess pollution reduction under demand shifted scenarios, Related to Figure 5 in the main text.*** *(Left plot) Excess load demand peak shifted 4 hours ahead of solar availability peak. (Middle plot) No demand shift. Same plot as Figure 13. (Right plot) Excess load demand peak delayed 4 hours relative to solar availability peak. Contour lines represent isolines of constant excess pollution reduction cost over the plotted surface.*

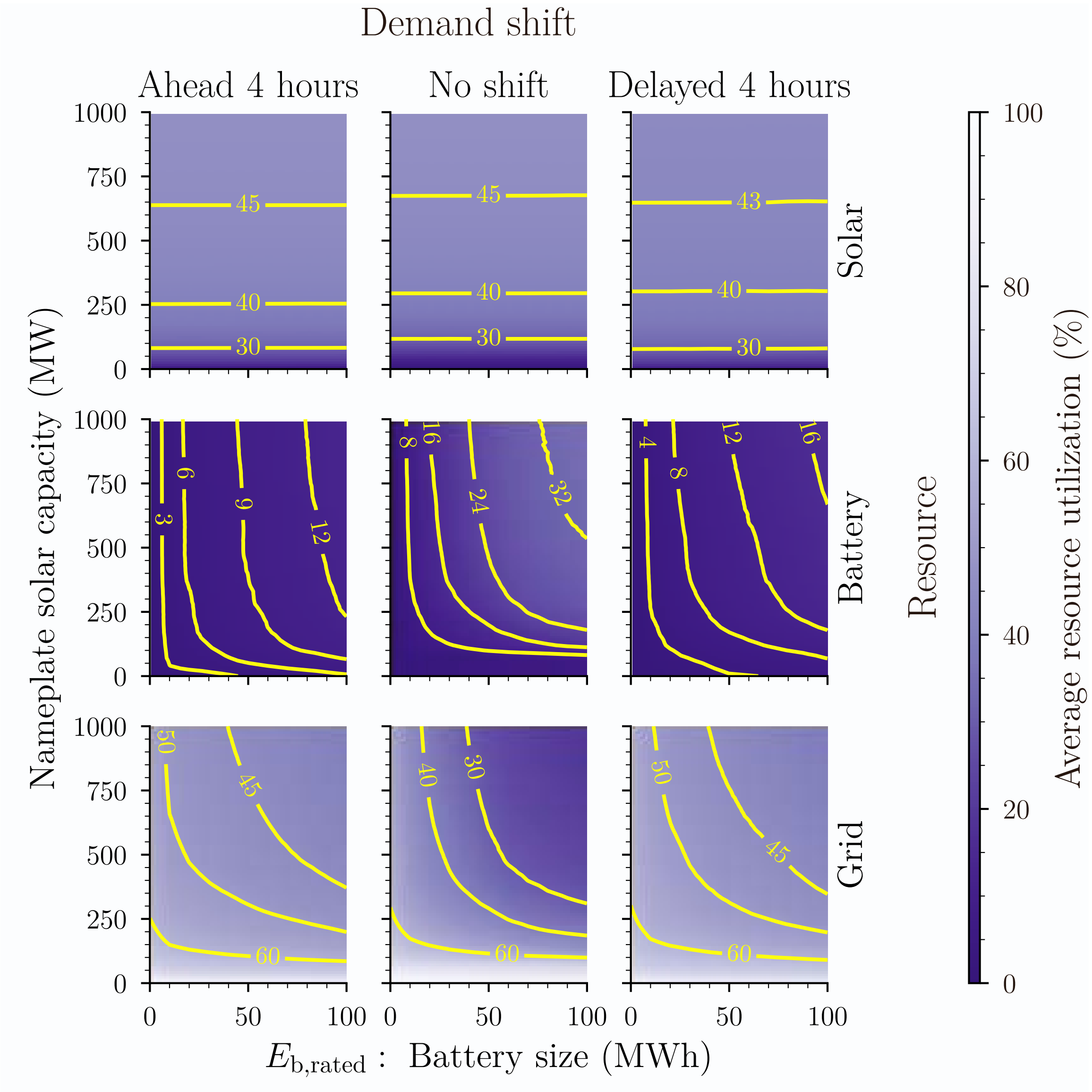

Figure S22: ***Resource utilization for different combinations of nameplate solar capacity and battery size for a hypothetical renewable energy site adjacent to Port of Savannah, Related to Figure 5 in the main text.*** *Each column: different demand shift scenarios. Each row: different battery chemistry. Contour lines represent isolines of constant average resource utilization over the different microgrid configurations.*

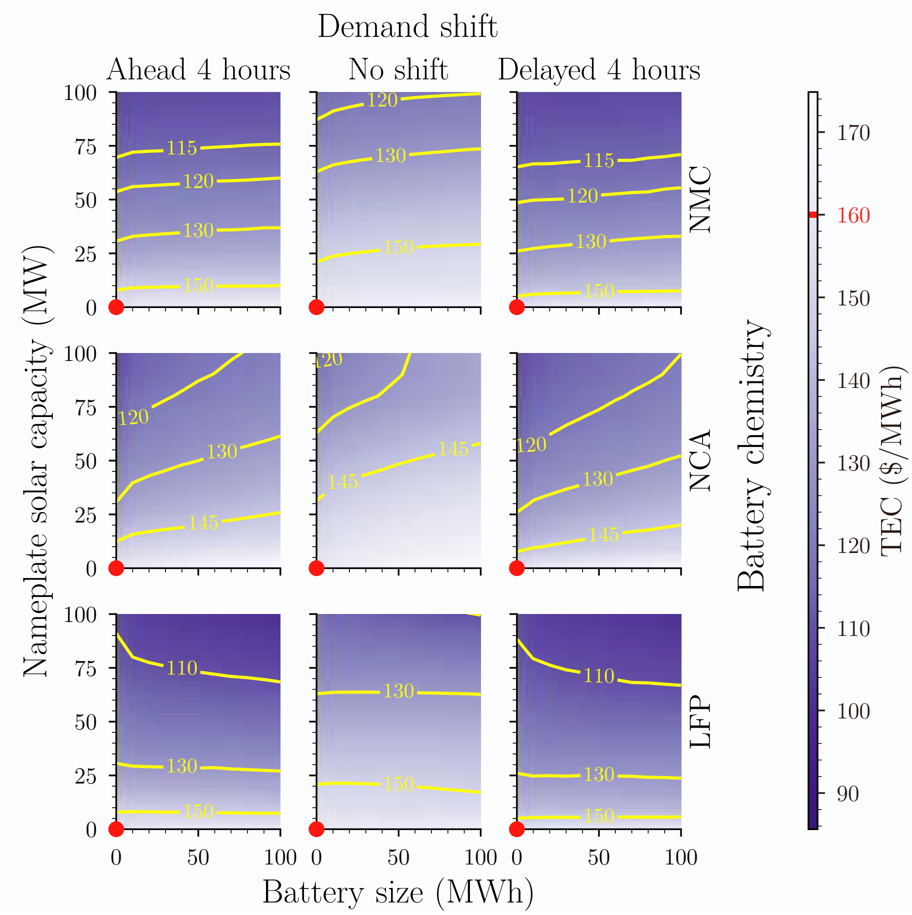

Figure S23: ***Total electricity cost (TEC) for different nameplate solar capacity and battery size combinations deployed at a hypothetical renewable energy site adjacent to the Port of Savannah, Related to Figure 7 in the main text.*** *Each column: different demand shift scenarios. Each row: different battery chemistry. Cost for a system with no solar and battery size is the grid electricity price of $160 /MWh (highlighted in red). Contour lines represent isolines of constant TEC over the different microgrid configurations.*

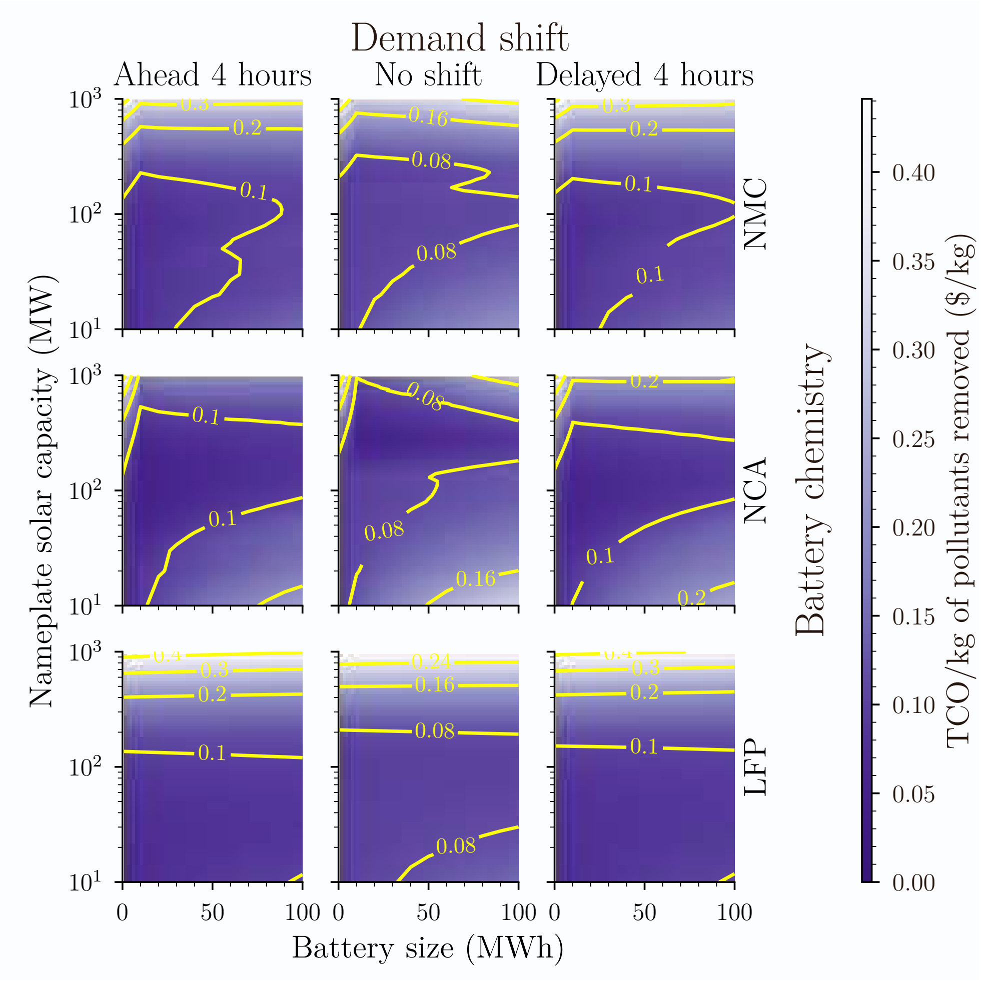

Figure S24: ***TCO/kg of removed for different combinations of nameplate capacity and battery size deployed at a hypothetical site adjacent to the Port of Savannah, Related to Figure 7 in the main text.*** *Each column: different demand shift scenarios. Each row: different battery chemistry. Contour lines represent isolines of constant utility metric over the different microgrid configurations*

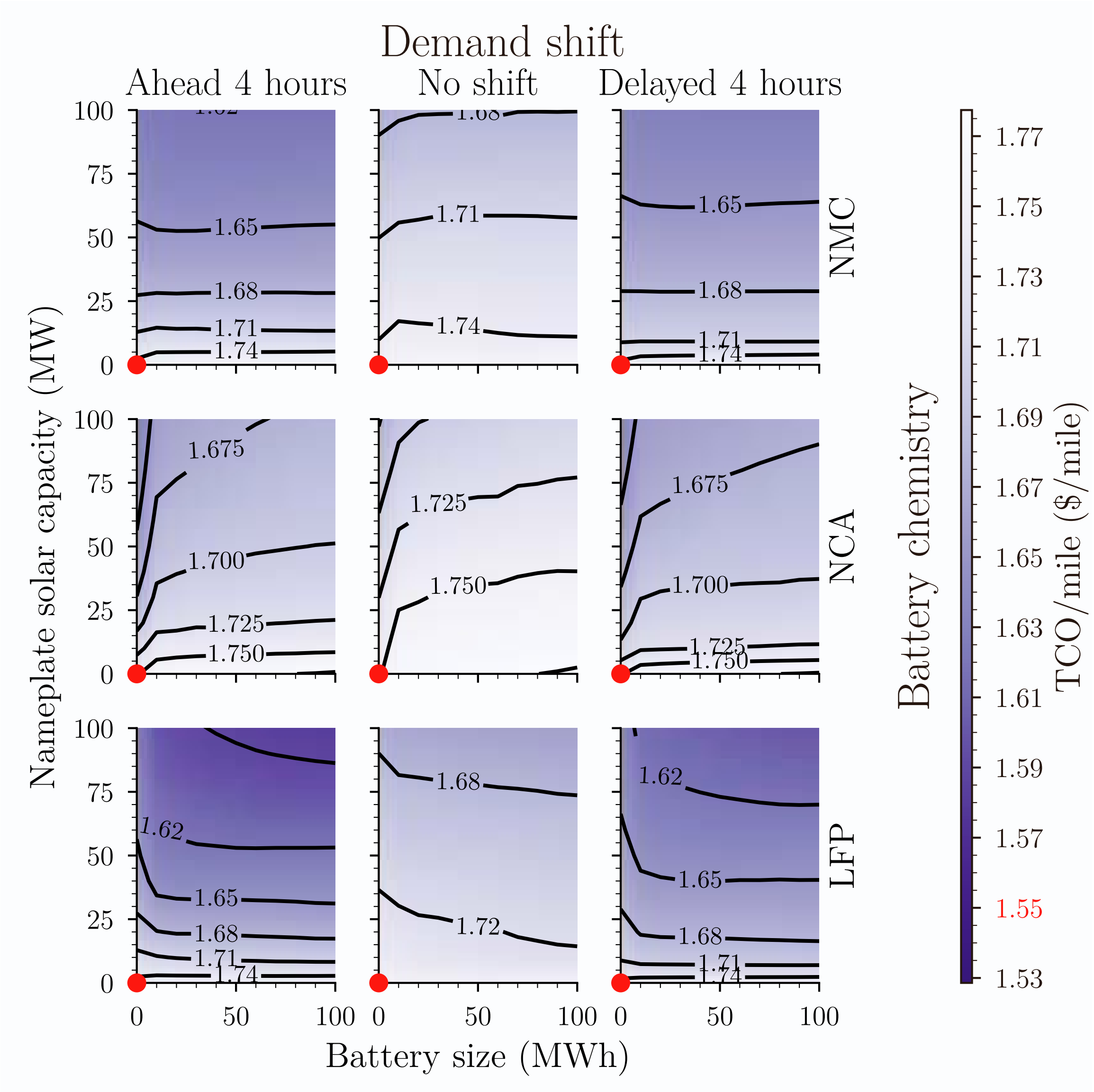

Figure S25: ***TCO/mile of the HDCV fleet for different combinations of nameplate capacity and battery size deployed at a hypothetical site adjacent to the Port of Savannah, Related to Figure 7 in the main text.*** *Each column: different demand shift scenarios. Each row: different battery chemistry. Baseline cost using only the grid to power the excess load demand is $1.55/mile. Contour lines represent isolines of constant TCO/mile over the different microgrid configurations*

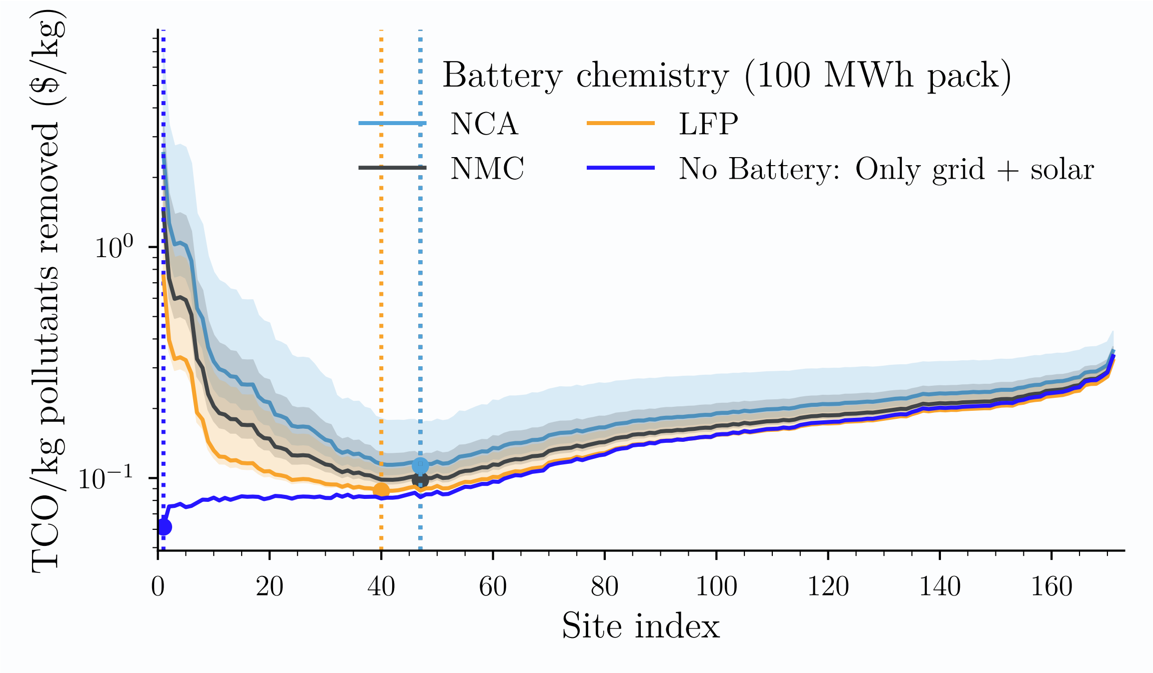

Figure S26: ***Utility cost metric, Related to Figure 7 in the main text. No battery: Microgrid deployment with only solar alone.*** *Other colors denote different choices of battery chemistry when a 100 MWh battery is deployed along with the solar installation.*

Table S1: Set of decision variables to minimize the objective and their interpretations

Variable [Unit]	Interpretation
$p_{\mathrm{g}}^{\ell}(\mathrm{t})$ [MW]	Power provided to load by grid
$p_{\mathrm{s}}^{\ell}(\mathrm{t})$ [MW]	Power provided to load by solar
$p_{\mathrm{b}}^{\ell}(\mathrm{t})$ [MW]	Power provided to load by battery
$p_{\mathrm{s}}^{\mathrm{b}}(\mathrm{t})$ [MW]	Power provided by solar to charge the battery
$p_{\mathrm{s}}^{\mathrm{curtail}}(\mathrm{t})$ [MW]	Curtailed solar power
$p_{\mathrm{g}}(\mathrm{t})$ [MW]	Total power pulled from the grid
$p^{\mathrm{b}}(\mathrm{t})$ [MW]	Charging power to the battery
$p_{\mathrm{g}}^{\mathrm{b}}(\mathrm{t})$ [MW]	Power from grid to charge battery
$E_{\mathrm{b}}(t)$ [MWh]	Energy of battery
$\delta_{\mathrm{b}}(t)$ [-]	Discharge indicator

Table S2: Generation module input parameters

Variable	Value	Meaning
system_capacity	1000	Power rating (kW) for system at each grid point
losses	14.07	Percent power lost due to external/internal inefficiencies
array_type	0 (Fixed tilt)	Designates whether to model solar tracking technology or rooftop mounted panels
azimuth	180	Module rotation from North (degrees)
dc_ac_ratio	1.3	Conversion factor between generated (DC) and distributed (AC) power ratings
gcr	0.4	Ratio between module surface area and land area occupied by the module
inv_eff	96	Inverter conversion efficiency between AC and DC power output
module_type	0 (Standard crystalline silicon)	Module type/crystal material to be modeled
tilt	latitude	Degrees tilted from horizontal. Latitude designates that tilt should increase with distance from equator.

Table S3: ***Summary of a 4-hour duration, 100 MWh battery pack characteristics used in this work. Related to Figure 6 and Figure 7 in the main text.***

Battery Chemistry	Rated power (MW)	Battery size (MWh)	Battery system voltage (V)	N_p	$N_{total,module}$	Cell capacity (Ah)	Cell nominal voltage (V)
NMC 811	25	100	811	4167	45837	14.80	3.68
NCA			808	4167	45837	14.86	3.67
LFP			794	4167	50004	15.11	3.31

Table S4: ***Battery pack cost estimate for a 100 MWh system for different choice of chemistries. Related to Figure 6 and Figure 7 of the main text.***

Chemistry	Battery system cost ($)	Battery system cost ($/kWh)
NMC 811	15,097,974	150.98
NCA	19,403,415	194.03
LFP	21,616,938	216.17

Table S5: ***Extra capital costs for battery system, Related to Figure 6 and Figure 7 of the main text. Items are evaluated in order from top to bottom.***

Item	Cost	Notes
Storage Balance of System	+ 23% of battery pack cost	This includes supporting cost components for the storage block with container, cabling, switchgear, flow battery pumps, and heating, ventilation, and air conditioning.
Power conversion system	45 $/kW (rated)	This component includes bidirectional invertor, DC-DC converter, isolation protection, alternating current breakers, relays, communication interface, and software. This is the power conversion system for batteries.
Controls & Communication	7.8 $/kW baseline cost for 10MW battery system. 50% reduction in cost from baseline going from 10 MW to 1 MW. 33% increase in cost from baseline going from 10 MW to 100 MW. Linear interpolation is used to obtain a cost for battery systems with power ratings between 10 and 100 MW.	This includes the energy management system for the entire battery system and is responsible for system operation. This may also include annual licensing costs for software. The cost is typically represented as a fixed cost scalable with respect to power and independent of duration.
System Integration	+ 5% of all items above	Price charged by the system integrator to integrate sub-components of a battery pack into a single functional system. This includes procurement and shipment to the site of battery modules, racks with cables in place, containers, and power equipment. At the site, the modules and racks are containerized with HVAC and fire suppression installed and integrated with the power equipment to provide a turnkey system.
Engineering, Procurement & Construction	+ 20% of all items above	This includes non-recurring engineering costs and construction equipment as well as shipping, siting and installation, and commissioning of the battery pack.
Project development	+ 20% of all items above	This includes costs associated with permitting, power purchase agreements, interconnection agreements, site control, and financing.
Grid integration	+ 1.5% of all items above	This includes the direct cost associated with connecting the ESS to the grid, including transformer cost, metering, and isolation breakers. For the last component, it could be a single disconnect breaker or a breaker bay for larger systems.

Table S6: ***Assumptions on cycle life for different Li-ion battery chemistries, Related to Table 2 of the main text.***

Battery chemistry	$N_{cycles}^{EOL\,(low)}$	N_{cycles}^{EOL}	$N_{cycles}^{EOL\,(high)}$
NMC	800	2000	2500
NCA	500	1400	2000
LFP	1800	6000	7000

Table S7: ***Model variable values for total cost of ownership calculation for electrified fleet, Related to Figure 7 of the main text.***

Variable	Value
MSRP ($/vehicle)	334,313
RC ($/vehicle)	1,500
SUB ($/vehicle)	40,000
INS ($/vehicle/year)	11,700
MAINT ($/mi)	0.055
$\boldsymbol{\eta}$	0.25

Table S8: ***Model variables for total cost of ownership for a pure diesel fleet, Related to Figure 7 of the main text.***

Variable	Value
MSRP ($/vehicle)	133,841
RC ($/vehicle)	1,500
SUB ($/vehicle)	0
INS ($/vehicle/year)	8,000
MAINT ($/mi)	0.086
$\boldsymbol{\eta}$	0.35

Table S9: ***Exclusion criteria, thresholds, and buffer distances for solar energy siting***

Criteria	Exclusion Threshold	Buffer
Population Density	>500 people per sq mi	20 miles
Protected Lands	None/No go	3 km
Wetlands and Open Water	None/No go	None
100-year Floodplains	None/No go	None
Landslide Hazards	None/No go	None
Slope	>5%	None
Major Roads	None/No go	None
Solar Radiation	<4.8 $kWh/m^2/day$	None

Methods S1

1. Quantifying the two problems with fleet electrification

This section provides an expanded discussion on how the excess load demand of an electrified HDCV fleet operating in the Port of Savannah region is estimated and how the associated increase in pollution intensity is quantified. We define the "Port of Savannah region" as the geographic area contained within the counties of Chatham, Jasper, Bryan, Liberty, and Effingham.

As described in the main text, using the OR-AGENT framework[1], an estimate of the hourly energy required by the full fleet of electrified trucks, shown in *Figure S1*, is generated.

This excess load demand is associated with an increase in pollutant intensity. The pollution intensity can be estimated by an approach first proposed in Sujan et al.[1]. Briefly, this approach integrates historical spatiotemporal data on grid load and pollution intensity across all U.S. counties and forecasts the pollution intensity associated with the excess load demand. Thus, given a county of interest, the county's historical electric load profiles, the historical energy generation profiles for that region, as well as the excess load profile, the excess pollution intensity can be estimated for every hour of the year. A schematic of the inputs and output of this pipeline is shown in *Figure S2*. By construction, when the excess load profile is zero, as it is on the weekend hours for example, the excess pollution intensity is also zero.

If additional energy resources are built that are as pollution intensive as the current grid infrastructure, the electrification of HDCVs may yield limited air quality improvements. For the Port of Savannah region, electrifying about 80% of the fleet with trucks that have an 800 kWh battery pack ensures full coverage of all the current routes; however, we estimate that this electrification will reduce pollution by only about 37.6% (see *Figure S3*) compared to a fully diesel-based fleet, if the same pollution-intensive energy generation sources used in the grid today are used to power the excess load demand.

2. Integration of utility-scale solar and battery power with the grid

We propose a renewable energy network consisting of solar and battery power to supplement grid resources (schematic shown in Figure 2 of the main text). In this section, we formally introduce the modeling framework that describes the power flows between network elements[2]. Here, we consider the excess load demand as being met by a combination of: (1) solar energy, originating from a single solar farm of a given nameplate capacity, (2) a utility-scale battery storage system, and (3) currently existing energy

resources, as well as to-be-built resources, that are not pollution-minimal which we label as the "grid".

2.1 Mathematical Formulation of the Renewable Energy Network

As mentioned in the main text, in our notation, for a power quantity denoted $p_i^j(t)$, the subscript i denotes the network element origin of the power and j denotes the target network element. An integer variable $t \in [1, 8760]$ is used to index the 8760 hours of a single year.

We denote the excess load demand from the electrified HDCVs by $p^\ell(t)$. The hourly available solar capacity is denoted as $p_\mathrm{s}(t)$. The available solar capacity can be used to provide power to the load $p_s^\ell(t)$, provide power to the battery $p_\mathrm{s}^\mathrm{b}(t)$, or be curtailed $p_\mathrm{s}^\mathrm{curtail}(t)$ if too much solar capacity is available:

$$p_\mathrm{s}(t) = p_\mathrm{s}^\ell(t) + p_\mathrm{s}^\mathrm{b}(t) + p_\mathrm{s}^\mathrm{curtail}(t) \qquad \text{(Equation 1)}$$

Similarly, the battery can be used to provide power $p_\mathrm{b}^\ell(t)$to meet the excess load demand. It may be recharged (via a charger) either through solar power $p_s^\mathrm{b}(t)$ or grid power $p_\mathrm{g}^\mathrm{b}(t)$. Thus, the charger power is

$$p^\mathrm{b}(t) = p_\mathrm{s}^\mathrm{b}(t) + p_\mathrm{g}^\mathrm{b}(t) \qquad \text{(Equation 2)}$$

The remaining energy stored in the LIB at a given hour $\boldsymbol{t}$ is modeled as,

$$E_\mathrm{b}(t) = E_\mathrm{b}(t-1) + \eta_\mathrm{chg} \cdot p^b(t) - \frac{1}{\eta_\mathrm{dchg}} p_\mathrm{b}^\ell(t) \qquad \text{(Equation 3)}$$

The battery energy increases upon charging and decreases upon discharging. The charging and discharging efficiencies are denoted as η_chg and η_dchg, respectively. The roundtrip efficiency $\boldsymbol{\mu}$ characterizes the battery system's performance and encapsulates the whole set of system losses. To simplify the model, and in accordance with the National Renewable Energy Lab's (NREL) Annual Technology Baseline [3], we use a roundtrip efficiency of $\mu = 0.85$ as being characteristic for Li-ion battery technologies. We do not distinguish between charging and discharging losses: We assume symmetric efficiencies, related to the roundtrip efficiency by $\eta_\mathrm{chg} = \eta_\mathrm{dchg} = \sqrt{\mu}$. The roundtrip efficiency generally varies between LIB chemistries/manufacturers; however, this variation is neglected here.

The existing grid resources can still be used as needed to meet the load demand and/or charge the battery. The power from the grid delivered to the load is denoted $p_\mathrm{g}^\ell(t)$ and the power from the grid used to charge the battery is $p_\mathrm{g}^\mathrm{b}(t)$. Thus, the total power generated by the grid is

$$p_{\mathrm{g}}(t) = p_{\mathrm{g}}^{\mathrm{b}}(t) + p_{\mathrm{g}}^{\ell}(t) \qquad \text{(Equation 4)}$$

The excess load demand in the renewable energy network is thus satisfied by a mix of solar, battery, and grid power according to

$$p^{\ell}(t) = p_{\mathrm{s}}^{\ell}(t) + p_{\mathrm{b}}^{\ell}(t) + p_{\mathrm{g}}^{\ell}(t) \qquad \text{(Equation 5)}$$

2.2 Optimal dispatch framework to maximally improve air quality

Given an estimate of the hourly available solar capacity $p_{\mathrm{s}}(t)$, and the excess load demand profile $p^{\ell}(t)$, we introduce the optimal dispatch framework used to determine how the grid and battery elements in the energy network are used. The dispatch is designed to maximize the yearly total air quality improvement while ensuring that the excess load demand at every hour of the year.

To do this, we first consider an energy network where only pollutant-intensive grid resources can be used to meet the excess load demand. This will be referred to as the *baseline network*. In the baseline network, the following relationship

$$p_{\mathrm{g}}(t) = p_{\mathrm{g}}^{\ell}(t) = p^{\ell}(t) \qquad \text{(Equation 6)}$$

between the total grid power and the excess load demand holds. The baseline network has an associated total pollutant intensity of

$$\varepsilon_{\mathrm{g}}^{\mathrm{base}} = \sum_{t=1}^{8760} \varepsilon_{\mathrm{g}}(t) \qquad \text{(Equation 7)}$$

where $\varepsilon_{\mathrm{g}}(t)$ denotes the hourly pollutant intensity (as estimated by OR-AGENT).

In contrast, the alternative energy network has an associated total excess pollutant intensity of

$$\varepsilon_{\mathrm{g}}^{\mathrm{alternative}} = \sum_{t=1,p^{\ell}(t)>0}^{8760} \frac{p_{\mathrm{g}}(t)}{p^{\ell}(t)} \cdot \varepsilon_{\mathrm{g}}(t) + \left[p_{\mathrm{s}}^{\ell}(t) + p_{\mathrm{s}}^{\mathrm{b}}\right] \cdot \varepsilon_{\mathrm{s}}(t) \qquad \text{(Equation 8)}$$

where the sum only contains hours where there is nonzero excess load demand. The first term in the sum represents the pollutant intensity from reduced grid usage when the microgrid is deployed. The second term represents the amount of life-cycle pollutant intensity associated with photovoltaics[4]. In this work it is set to $\varepsilon_{s}(t) = 0.048$ US ton of pollutants generated/MW of photovoltaic usage.

An optimization problem is formulated to obtain the optimal dispatch of the three energy sources in the network with the objective being to maximize the percentage of *unrealized air quality improvement* of the alternative energy network,

$$J[\Theta] = 100 \times \left(\frac{\varepsilon_{\mathrm{g}}^{\mathrm{alternative}}}{\varepsilon_{\mathrm{g}}^{\mathrm{base}}} - 1 \right) . \qquad \text{(Equation 9)}$$

This is accomplished by varying, within their respective bounds, the set of decision variables

$$\Theta = \left\{ p_{\mathrm{g}}^{\ell}(t), p_{\mathrm{s}}^{\ell}(t), p_{\mathrm{b}}^{\ell}(t), p_{\mathrm{s}}^{\mathrm{b}}(t), p_{\mathrm{s}}^{\mathrm{curtail}}(t), p_{g}(t), p^{b}(t), p_{\mathrm{g}}^{\mathrm{b}}(t), E_{b}(t), \delta_{b}(t) \right\} ,$$

some of which were introduced in the main text, are recapitulated in *Table S1*.

By convention, we set a lower bound for the power flow quantities

$$p_{\mathrm{g}}^{\ell}(t), p_{\mathrm{s}}^{\ell}(t), p_{\mathrm{b}}^{\ell}(t), p_{\mathrm{s}}^{\mathrm{b}}(t), p_{\mathrm{s}}^{\mathrm{curtail}}(t), p_{g}(t), p^{b}(t), p_{\mathrm{g}}^{\mathrm{b}}(t) \geq 0 . \qquad \text{(Equation 10)}$$

The battery system is assumed to start the year half full,

$$E(0) = 0.5 \cdot E_{\mathrm{b,rated}} , \qquad \text{(Equation 11)}$$

where the nominal energy of the battery system (which we will refer to as the *battery size*) is denoted $E_{\mathrm{b,rated}}$. To enable repeated yearly dispatch analysis, we impose that

$$E(8760) = E(0) \qquad \text{(Equation 12)}$$

thereby, allowing us to straightforwardly extrapolate the same battery dispatch to subsequent years[1]. Additionally, to maximize lifetime the battery's energy is limited to the energy range

$$0.1\, E_{\mathrm{b,rated}} \leq E_{\mathrm{b}}(t) \leq 0.9\, E_{\mathrm{b,rated}} \qquad \text{(Equation 13)}$$

Moreover, the battery is unable to charge and discharge at the same time. When it does (dis)charge, it can only do so up to a specified maximum power set by the duration of the battery and the battery size

$$\tau_{\mathrm{b}} = \frac{E_{\mathrm{b,rated}}}{p_{\mathrm{b,max}}} \qquad \text{(Equation 14)}$$

In this work, we consider batteries with a 4-hour ($\tau_{b} = 4$) duration, as this duration currently serves as the de-facto limit for a large majority of deployed utility-scale battery

---

[1] This assumes that the excess load demand $p^{\ell}(t)$ and the available solar capacity profile $p_{\mathrm{s}}(t)$ also do not change in subsequent years.

systems [5]. These restrictions on the battery behavior can be summarized in the following relations:

$$p_{\mathrm{b}}^{\ell}(t) \leq \delta_{\mathrm{b}}(t) \cdot p_{\mathrm{b,max}} \qquad \text{(Equation 15a)}$$

$$p^{\mathrm{b}}(t) \leq \left(1 - \delta_{\mathrm{b}}(t)\right) \cdot p_{\mathrm{b,max}} \qquad \text{(Equation 15b)}$$

A binary decision variable $\delta_{\mathrm{b}}(t) \in \{0,1\}$ is introduced that indicates if the battery is discharging ($\delta_{\mathrm{b}}(t) = 1$) or charging ($\delta_{\mathrm{b}}(t) = 0$).

The grid power is restricted such that no power is pulled from the grid when excess load demand is zero,

$$p_{\mathrm{g}}(t) = 0 \;\; \forall\, t \mid p^{\ell}(t) = 0 \qquad \text{(Equation 16)}$$

The optimal dispatch of the alternative energy network is thus the set of decision variables Θ^* that satisfy

$$\Theta^* = \underset{\Theta}{\operatorname{argmax}}\, J[\Theta] \qquad \text{(Equation 17)}$$

subject to the Equations 1 to 5 and constraints Equations 10 to 17. This optimization is a mixed-integer linear program (MILP), consistent with optimal dispatch frameworks explored previously in literature[6–8]. The MILP is solved in MATLAB using the **intlinprog** function in MATLAB's Optimization Toolbox.

2.3 Average resource utilization of resources to power excess load demand

Having obtained the optimal set of powers $p_g^{\ell}(t)$ (grid), $p_s^{\ell}(t)$ (solar), and $p_b^{\ell}(t)$ (battery) that meets the excess load demand $p^{\ell}(t)$ from the optimization procedure outlined in Section 2.2, we compute the average utilization of each resource, respectively, by

$$f^{\mathrm{grid}} = \frac{1}{N_{p^{\ell}(t)>0}} \sum_{t=1,p^{\ell}(t)>0}^{8760} \frac{p_{\mathrm{g}}^{\ell}(t)}{p^{\ell}(t)} \qquad \text{(Equation 18a)}$$

$$f^{\mathrm{solar}} = \frac{1}{N_{p^{\ell}(t)>0}} \sum_{t=1,p^{\ell}(t)>0}^{8760} \frac{p_{\mathrm{s}}^{\ell}(t)}{p^{\ell}(t)} \qquad \text{(Equation 18b)}$$

$$f_{\mathrm{dchg}}^{\mathrm{batt}} = \frac{1}{N_{p^{\ell}(t)>0}} \sum_{t=1,p^{\ell}(t)>0}^{8760} \frac{p_{\mathrm{b}}^{\ell}(t)}{p^{\ell}(t)} \qquad \text{(Equation 18c)}$$

where $N_{p^{\ell}(t)>0}$ denotes the number of hours in a year where the excess load demand is nonzero. The average utilization of each resource to power the excess load demand sums to unity:

$$f^{\text{grid}} + f^{\text{solar}} + f_{\text{dchg}}^{\text{batt}} = 1 \quad \text{(Equation 19)}$$

2.4 Fraction of charging resources

Like calculations performed in Section 2.3, we compute the average utilization of grid and solar resources for battery charging. These can be estimated by the following relations, respectively:

$$f_{\text{chg}}^{\text{grid}} = \frac{1}{N_{p^{\text{b}}(t)>0}} \sum_{t=1,p^{\text{b}}(t)>0}^{8760} \frac{p_{\text{g}}^{\text{b}}(t)}{p^{\text{b}}(t)} \quad \text{(Equation 20a)}$$

$$f_{\text{chg}}^{\text{solar}} = \frac{1}{N_{p^{\text{b}}(t)>0}} \sum_{t=1,p^{\text{b}}(t)>0}^{8760} \frac{p_{s}^{b}(t)}{p^{b}(t)} \quad \text{(Equation 20b)}$$

As before, the average utilization of each resource for charging sums to unity:

$$f_{\text{chg}}^{\text{grid}} + f_{\text{chg}}^{\text{solar}} = 1 \quad \text{(Equation 21)}$$

3. Siting framework for photovoltaic resources

To reduce the overall reliance on fossil fuels and lower the risk of grid failure, it is necessary to supplement the existing power grid with alternative energy resources (RES). Modeling the potential for siting and generation of these resources is carried out here by integrating two modeling technologies: the OR-SAGE tool from ORNL and the Renewable Energy Potential (reV) model from the National Renewable Energy Laboratory (NREL)[9].

3.1 Regional solar viability assessment

The assessment of solar viability in this section is performed using a framework known as the Oak Ridge Siting Analysis for Generation Expansion (OR-SAGE)[10,11]. As discussed in the main text, OR-SAGE makes use of various high-resolution geospatial data layers that integrate existing restrictions on land use based on various factors. While some layers directly correspond to whether a given region is suitable for siting of a given technology, other layers, such as layers describing variations in slope, must be converted into a decision layer by using a filtering threshold on the data. These thresholds are generally determined from safety requirements or from constraints created by the technology under consideration. The set of data layers that are used in this work to assess the regional siting

of solar energy, along with their associated thresholds and buffer zones, are detailed in *Table S9*.

Within each decision layer, a value of 0 indicates that a grid square is suitable for siting, and a value of 1 indicates a violation of the constraint specific to that layer. These data layers can then be overlayed to create a composite siting map, where the value of a grid square corresponds to the number of constituent data layers that conflict with a siting decision at the given location. The resulting composite map, obtained from cross-referencing the various solar energy siting layers, can be seen in *Figure S4*.

For this analysis, the region of Chatham County, Georgia is selected for its proximity to the Port of Savannah. The three counties surrounding Chatham are also included in this analysis to encompass all available areas within the county. The section of the solar viability map corresponding to this region can be seen in *Figure S5*.

3.2 Hourly available solar capacity assessment per possible site

For this analysis, the 2019 National Solar Radiation Database (NSRDB, v3) is used to estimate the hourly available capacity at each site. Once the region of interest (ROI) is established, the configuration of the solar technology is specified. The system configuration parameters and values selected for this analysis are listed in *Table S2*. These values reflect a relatively inexpensive configuration, utilizing standard crystalline silicon photovoltaic (PV) cells and a fixed tilt mounting system.

These parameters, along with the selected NSRDB points, are passed on to reV's generation module. The generation module is then used to calculate capacity factor values——the ratio between the actual generation capacity of a site and its nameplate capacity——for a theoretical solar farm placed at each selected grid-point in the NSRDB dataset. This is done for every hour in the chosen year, which results in a set of temporal capacity factor profiles for each farm. The average capacity factors for Chatham Georgia and the surrounding counties in 2019 can be seen in *Figure S6*.

The representative profile module of reV aggregates the capacity factor profiles created in the generation module to the same resolution as the parcels in the supply curve aggregation module. Here, the default 'meanoid' method in reV is used to generate the representative profiles for each supply curve point. Post-processing is performed on the reV output to calculate the hourly available capacity profiles $p_s(t)$ from representative capacity factors and nameplate generation capacities. In *Figure S7*, the available solar capacity for three of the 171 viable sites, representing a site with low, median, and high nameplate solar capacity are shown.

4. Estimating costs of deployment

This section details on the cost of deployment for a microgrid integrating solar and battery systems, from the perspectives of different stakeholders in freight transportation. The capital cost estimates of the solar (Section 4.1) and battery systems (Section 4.2) are each estimated separately, and additional costs are included to capture integration and deployment. The trends in the total capital costs are discussed in Section 4.3. The total cost of ownership framework is then mathematically formulated in Section 4.4. Given this TCO framework, two separate cost metrics for deployment are assessed for the utility and the fleet operator, in Sections 4.5 and 4.6, respectively.

4.1 Capital cost expenses for photovoltaic farms

As described in the main text, we have developed a comprehensive capital cost calculator based on the U.S. solar PV system benchmarks reported by NREL in their PVSCM model[12]. The capital cost of the solar PV system is divided into two main categories: equipment cost and development cost.

The equipment cost comprises various components as shown in *Figure S8*:

- The module includes photovoltaic cells, which convert sunlight into electricity, and the frame, which provides structural support. Additional elements are the junction box, electrical connections, and assembly materials.
- Inverters include power devices that convert DC to AC power, the rest of the inverter system, and packaging materials for shipping and protection.
- The structural and balance of system (SBOS) includes torque tubes, driven piers, module rails, fasteners, slew drives for rotation, dampers to reduce vibrations, motors for tracking, and control electronics for PV operations.
- The electrical balance of system (EBOS) consists of transformers, switches, breakers, conductors, combiner boxes, grounding systems, substations, transmission lines, and network upgrades to enhance existing electrical infrastructure.

The development cost includes fieldwork and office work components. Fieldwork encompasses labor for installation, site preparation, inspection, and construction. Office work involves permitting, engineering and design, project management, contingency planning, and interconnect and communication.

Summing these costs yields an estimate of the total overnight capital cost of the solar farm shown in *Figure S9*. Intuitively, the cost is monotonic with nameplate solar capacity; nevertheless, even the smallest nameplate solar capacity site corresponding to 1 MW

incurs over $1 million to build and install while the largest sites that we consider approach $1 billion.

4.2 Capital cost estimates for energy storage technologies

To estimate the capital cost variation of different LIB systems, we leverage the capabilities of the Battery Performance and Cost (BatPaC Version 5.1) tool, developed by Argonne National Lab[13]. We estimate, in Section 4.2.1, the capital costs for building a battery pack using the leading three chemistries[2] currently used in the industry: NMC (811), NCA, and LFP[14]. Subsequently, the capital costs associated with installation and deployment are estimated in Sec. 4.2.2 leveraging a report from the Pacific Northwest National Laboratory (PNNL).

4.2.1 BatPaC capital cost estimation for battery pack

To obtain a capital cost estimate for the battery pack using a given chemistry, BatPaC requires the user to specify the details of the battery pack. We create battery pack in BatPaC with a rated duration of 4 hours, obtained by appropriately specifying the target power and the rated energy of the pack. As utility-scale storage is a long-duration application, we consider only energy-type cells in our analysis. The maximum positive electrode thickness is set to 80 micrometers while the maximum cell thickness is set to 25 millimeters. For the pack configuration, the number of modules in parallel[3] is set to 2, the number of modules in a row is set to 10, and the number of rows of modules is set to 4. Fixing these settings, the number of cells in parallel within a module is determined from

$$N_{\mathrm{p}} = \frac{1}{2}\left\lceil \frac{E_{\mathrm{b,rated}}}{V_{\mathrm{pack,target}} \cdot Q_{\mathrm{cell,target}}} \right\rceil \quad \text{(Equation 22)}$$

where $V_{\mathrm{pack,target}} = 800\ V$ and $Q_{\mathrm{cell,target}} = 15\ Ah$ are the target pack voltage and cell capacity, respectively. The factor of ½ comes from the 2 modules that are connected in parallel. Correspondingly, the total number of cells in a module is computed by,

$$N_{\mathrm{total,module}} = \frac{\left(\frac{V_{\mathrm{pack,target}}}{V_{\mathrm{cell,nominal}}}\right)}{\left(\frac{10 \cdot 4}{2}\right)} \cdot N_p \quad \text{(Equation 23)}$$

[2] For the lithium-ion batteries we consider, we assume that the negative electrode material is always lithiated graphite. As such, here "chemistry" in this work refers to a choice of positive electrode material. Other choices could be made for the negative electrode which could constitute a different "chemistry".

[3] This is a limitation of BatPaC that it becomes more inaccurate in estimating the quantity of connecting equipment necessary if the number of modules connected in parallel is set to more than 2.

We denote the nominal cell voltage (found after optimization in BatPaC) by $V_{\mathrm{cell,nominal}}$. The denominator accounts for the number of modules connected in series. A summary of the characteristics for a 100 MWh battery pack for different chemistries is given in *Table S3*. We find that packs using NMC 811 or NCA battery cells are configured to the same configuration, owing to the similarity in their cell-level characteristics, particularly their nominal voltage. As the nominal voltage of each LFP cell is lower than that of an NMC 811 or NCA cell, the LFP battery pack requires more cells to meet the required target voltage.

BatPaC allows different assumptions related to the pack manufacturing. In our analysis, we make the following assumptions:

1. There is a single gigafactory dedicated to producing the grid-scale storage pack.
2. The energy throughput of this gigafactory is fixed to 35 GWh/year.
3. The cell yield is 95%.
4. Any additional costs in fitting the factory for a specific size of battery pack are neglected. (i.e., it is assumed that the factory can be retooled from producing 1 MWh battery packs to 50 MWh packs without incurring any additional costs)

These assumptions ultimately set a lower limit on the costs of the battery pack. Except for the thermal management system costs, which we remove from the BatPaC analysis (this cost is included in the storage balance of systems instead, see Section 4.2.2), default values included in BatPaC are generally used for this analysis. The cost of a 100 MWh battery pack for a given chemistry generated from this process is summarized in *Table S4*.

4.2.2 ESS system installation and integration costs

Having estimated the cost of the battery pack, we now incorporate the costs associated with installation and integration of the battery pack to the grid to estimate the overall battery system cost. We use a report[15] from PNNL released in 2022, to estimate the associated costs of installation and integration of the battery system. The additional cost items, their values, and descriptions are summarized in *Table S5*.

The total battery system cost is shown in *Figure S10*. Intuitively, as the battery system size (i.e. the system nominal energy) increases, the greater the cost becomes. Interestingly, the cost of NCA and LFP battery systems is greater than that of the solar installation, while NMC is relatively similar. Nevertheless, in raw capital cost, NMC is estimated to provide the lowest capital cost of all the chemistries considered.

4.3 Total overnight capital cost

The total overnight capital cost of deployment for all different combinations of nameplate solar capacities and battery sizes, respectively, is shown in *Figure S11*. As expected from

their separate trends, we find that the most expensive systems are those with the largest nameplate solar capacity site and the largest battery size.

4.4 Total cost of ownership framework

In this section, we mathematically formulate the total cost of ownership framework, described in the main text. Here, we take the total cost of ownership (TCO) to be,

$$\mathrm{TCO} = \mathrm{IC} + \mathrm{AOC} \cdot \mathrm{YrsOp}\,, \qquad \text{(Equation 24)}$$

where IC ($) is the initial costs, AOC ($/year) is the annual operating cost, and YrsOp is the number of years of operation considered. The costs comprising IC and the AOC vary depending on the stakeholder of interest.

Two metrics must be defined to quantify the deployment and operational cost of the microgrid system: (1) the levelized cost of photovoltaic recharge, and (2) the levelized cost of storage. We now describe each of these quantities and their computation in turn.

The levelized cost of photovoltaic recharge, denoted as LCOPR ($/MWh), quantifies the cost of discharging a given solar resource[16] and is defined as

$$\mathrm{LCOPR} = \frac{\mathrm{OCC_{PV}}}{\mathrm{YrsOp_{PV}} \cdot E_{\mathrm{y,PV}}^{\mathrm{out}}} \qquad \text{(Equation 25)}$$

where $\mathrm{OCC_{PV}}$ ($) is the overnight capital cost of the solar installation, $\mathrm{YrsOp_{PV}}$ (year) is the number of years that the solar plant expects to operate, and $E_{\mathrm{y,PV}}^{\mathrm{out}}$ (MWh/year) is the total energy output of a given solar installation/site over a single year

$$E_{\mathrm{y,PV}}^{\mathrm{out}} = \sum_{t=1}^{8760} p_{\mathrm{s}}(t) \qquad \text{(Equation 26)}$$

The denominator of Eq. (25) is thus the total energy output of the solar farm throughout its lifetime. To simplify our analysis, we neglect any degradation in the performance of the solar plant over its lifetime.

The levelized cost of storage, denoted as LCOS ($/MWh), quantifies the cost of discharging the battery system and is defined by

$$\mathrm{LCOS} = \frac{\mathrm{OCC_b}}{N_{\mathrm{cycles}}^{\mathrm{EOL}} \cdot E_{\mathrm{b,rated}}} \qquad \text{(Equation 30)}$$

where OCC_{b} ($) is the overnight capital cost of the battery system installation,

The denominator of Equation 27 thus measures the total amount of discharged energy by the battery system throughout its life. The values used for the number of end-of-life cycles for each Li-ion battery chemistry are informed by manufacturer specification sheet data collected in[17], assume a depth-of-discharge of 80%, and listed in *Table S6*.

The LCOS for different battery sizes and for different chemistry choices are shown in *Figure S12*. The average value for each curve is reported in Table 2 of the main text. Due to its high expected lifetime, the LCOS of LFP is lower than the other chemistries despite its high capital cost. In contrast, NCA has the highest LCOS of all 3 chemistries due to its high capital cost and low expected lifetime.

Using these metrics, and with the utilization of each resource used meet the excess load demand that was computed in Section 2.3, the TEC is formulated as

$$\mathrm{TEC} = f^{\mathrm{solar}} \cdot \mathrm{LCOPR} + f_{\mathrm{dchg}}^{\mathrm{batt}} \cdot (\mathrm{COC} + \mathrm{LCOS}) + f^{\mathrm{grid}} \cdot \mathrm{GEP} \qquad \text{(Equation 31)}$$

Here, $\mathrm{GEP} = 160$ ($/MWh) for the Port of Savannah region represents the electricity price associated with the existing grid, aligned with prices reported by the Bureau of Labor Statistics for the Atlanta, GA region[18]. COC ($/MWh) is the cost of charging that is computed by

$$\mathrm{COC} = f_{\mathrm{chg}}^{\mathrm{solar}} \cdot \mathrm{LCOPR} + f_{\mathrm{chg}}^{\mathrm{grid}} \cdot \mathrm{GEP} \qquad \text{(Equation 32)}$$

where the average utilization of solar and grid for charging, $f_{\mathrm{chg}}^{\mathrm{solar}}$ and $f_{\mathrm{chg}}^{\mathrm{grid}}$, are defined in Section 2.4. Having defined the TEC, we now turn to defining the costs for two different stakeholders: the utility and the fleet operator

4.4.1 The cost to the utility

In this section, we introduce a cost metric for the utility: the TCO per kilogram of excess pollution mitigated. This metric is designed to incentivize utilities to supply power for the electrified fleet while using lower-pollution resources. Without such an incentive, utilities may resort to deploying pollutant-intensive resources to meet the increased demand. Thus, this metric quantifies the balance for utilities between the financial cost of deployment and their effectiveness in utilizing alternative energy technologies. By doing so, it ensures that the environmental benefits of electrifying heavy-duty vehicles are fully realized.

To compute this metric, we first estimate the TCO of solar and battery system deployment, as relevant to the utility. For the utility, the initial costs ($\mathrm{IC}_{\mathrm{utility}}$, $) will be the overnight capital costs of deployment for both solar, OCC_{PV}, and the battery systems, $\mathrm{OCC}_{\mathrm{b}}$:

$$\mathrm{IC_{utility}} = \mathrm{OCC_{PV,tot}} + \mathrm{OCC_{b,tot}} \qquad \text{(Equation 33)}$$

Since the utility-scale PV system and the battery system have different lifetimes, we formulate the cost based on the longer-lived component, accounting for the necessary replacements of the shorter-lived component. Thus, we define the total overnight capital cost of the PV system as:

$$\mathrm{OCC_{PV,tot}} = \left(\frac{\mathrm{YrsOp_{max}}}{\mathrm{YrsOp_{PV}}}\right) \cdot \mathrm{OCC}_{PV} \qquad \text{(Equation 27)}$$

with the overnight capital cost of a fresh utility-scale PV system denoted as $\mathrm{OCC_{PV}}$ (see Section 4). Similarly, the total overnight capital cost of the battery system is

$$\mathrm{OCC_{b,tot}} = \left(\frac{\mathrm{YrsOp_{max}}}{\mathrm{YrsOp_{b}}}\right) \cdot \mathrm{OCC}_{b} \qquad \text{(Equation 28)}$$

The system's total number of years of operation, from the utility's perspective, are taken to be the maximum number of years before either the solar or the battery system reaches its end of life. For solar, the number of years of operation is set to be 30 years[19]. For the battery's lifetime, we compute the number of years required before the end-of-life number of cycles is reached. To do this, from the battery dispatch signal we estimate how many cycles N_y^{cycles} the battery goes through each year of operation by the cycle counting algorithm detailed in[20]. The number of years of operation for the battery depends on chemistry according to

$$\mathrm{YrsOp_b} = \frac{N_{\mathrm{cycles}}^{\mathrm{EOL}}}{N_y^{\mathrm{cycles}}} \qquad \text{(Equation 28)}$$

where $N_{\mathrm{cycles}}^{\mathrm{EOL}}$ (-) is the number of cycles that the battery can undergo before the system's end-of-life. Thus, the overall lifetime of the system is

$$\mathrm{YrsOp_{max}} = \max(30, \min(15, \mathrm{YrsOp_b})) \qquad \text{(Equation 29)}$$

where 30 years is the lifetime of a typical PV system and 15 represents the maximum number of years that a battery system is expected to last given calendar aging[15], even if the maximum number of cycles has not been reached yet.

The utility bears the maintenance cost of the microgrid which forms its annual operating cost,

$$\mathrm{AOC_{utility}} = \mathrm{MAINT_{utility}} \qquad \text{(Equation 34)}$$

In line with previous work of[15] we represent the yearly maintenance cost of both the solar and the battery as a fraction of their respective overnight capital cost

$$\mathrm{MAINT_{utility}} = 0.02 \cdot \mathrm{OCC_{PV,tot}} + 0.004 \cdot \mathrm{OCC_{b,tot}} \qquad \text{(Equation 35)}$$

where the fractions are obtained from[12] for solar and[15] for LIBs.

Given the total excess load demand per year

$$E_{\mathrm{total}}^{\ell} = \sum_{t=1}^{8760} p^{\ell}(t) \qquad \text{(Equation 36)}$$

the annual revenue of the utility is defined to be,

$$\mathrm{REV_{utility}} = \mathrm{TEC_{utility\,<-fleet}} \cdot \left(E_{\mathrm{total}}^{\ell} - \sum_{t=1}^{8760} p_{\mathrm{g}}(t) \right) + \mathrm{MAINT_{utility}} \qquad \text{(Equation 37)}$$

The term in the brackets denotes the amount of energy generated by the microgrid (solar + battery). The $\mathrm{TEC_{utility\,<-fleet}}$ represents the total cost of electricity ($/MWh) that is associated with the microgrid that is received by the utility from the fleet and is designed such that, over the lifetime of the microgrid system, the total initial costs are recovered

$$\mathrm{TEC_{utility\,<-fleet}} = \frac{\mathrm{IC_{utility}}}{YrsOp_{max} \cdot \left(E_{total}^{\ell} - \sum_{t=1}^{8760} p_g(t) \right)} \qquad \text{(Equation 39)}$$

The yearly maintenance cost $MAINT_{utility}$ is directly passed from the utility to the fleet.

We note that the estimate of Eq. (33) will generally overestimate the lifetime of the battery system as it neglects any calendar aging effects[21,22]. As such, this analysis gives a lower bound on the costs to the utility. Future work will seek to improve the life models of the batteries and improve the estimates for the TCO to the utility. The utility TCO for a solar + battery system is therefore

$$\mathrm{TCO_{utility}} = \mathrm{IC_{utility}} + \mathrm{AOC_{utility}} \cdot \mathrm{YrsOp_{fleet}} - \mathrm{REV_{utility}} \cdot \mathrm{YrsOp_{fleet}} \qquad \text{(Equation 40)}$$

The amount of pollutants removed, measured in kilograms, is computed by the difference of the total pollutants of the alternative energy network and the baseline network

$$W_{\mathrm{poll}} = \varepsilon_{\mathrm{g}}^{\mathrm{alternative}} - \varepsilon_{\mathrm{g}}^{\mathrm{base}} \qquad \text{(Equation 41)}$$

obtained from the optimization detailed in Section 2.2.

4.4.2 The cost per mile to the fleet operator

To quantify the cost of using the alternative energy network to the fleet operator, we use the TCO of an HDCV per mile that it is driven, computed for the full fleet. For each vehicle in the fleet, the initial cost is computed by the sum of the Manufacturer's Suggested

Retail Price (MSRP, $/vehicle) and the registration cost (RC, $/vehicle) less the subsidies (SUB, $/vehicle) of the government. Assuming a uniform initial cost for all $N_{\mathrm{veh,BEV}} = 1631$ vehicles in the fleet, the total initial cost of the BEV portion of the fleet is

$$\mathrm{IC}_{\mathrm{fleet,BEV}} = N_{\mathrm{veh,BEV}} \cdot (\mathrm{MSRP}_{\mathrm{BEV}} + \mathrm{RC}_{\mathrm{BEV}} - \mathrm{SUB}_{\mathrm{BEV}}) \quad \text{(Equation 42)}$$

Simiarly, the diesel portion ($N_{\mathrm{veh,diesel}} = 460$) of the fleet is

$$\mathrm{IC}_{\mathrm{fleet,diesel}} = N_{\mathrm{veh,diesel}} \cdot (\mathrm{MSRP}_{\mathrm{diesel}} + \mathrm{RC}_{\mathrm{diesel}} - \mathrm{SUB}_{\mathrm{diesel}}) \quad \text{(Equation 43)}$$

As with the utility case (Section 4.4.1), the total operating cost of the fleet can be computed as the product of the annual operating costs multiplied by the number of years of operation. The annual operating costs of the fleet depends on the insurance costs per truck in the fleet (INS, $/vehicle), paid every year, the maintenance cost of the fleet (MAINT, $/mile), and the amount of electricity that the fleet uses quantified by the TEC:

$$\mathrm{AOC}_{\mathrm{fleet,BEV}} = \mathrm{INS}_{\mathrm{diesel}} \cdot N_{\mathrm{BEV}} + \mathrm{MAINT}_{\mathrm{BEV}} \cdot \mathrm{VMT}_{\mathrm{BEV,tot}} + \mathrm{TEC} \cdot E^{\ell}_{\mathrm{total}} + \mathrm{MAINT}_{\mathrm{utility}} \quad \text{(Equation 44)}$$

The annual operating cost for the diesel portion of the fleet is

$$\mathrm{AOC}_{\mathrm{fleet,diesel}} = \mathrm{INS}_{\mathrm{diesel}} \cdot N_{\mathrm{diesel}} + \mathrm{MAINT}_{\mathrm{diesel}} \cdot \mathrm{VMT}_{\mathrm{diesel,tot}} + C_{\mathrm{fuel}} \cdot \mathrm{VMT}_{\mathrm{diesel,tot}} \quad \text{(Equation 45)}$$

The amount of vehicle miles travelled by all the vehicles in the fleet per year is denoted by $\mathrm{VMT}_{\mathrm{tot}}$. The number of years of operation for the electrified HDCVs is taken to be 5 years, $\mathrm{YrsOp} = 5$. Note the correspondence between the last term in the AOC of the fleet (Equation 38) and the AOC of the utility (Equation 32): The energy provided by the utility is used by the fleet. The residual value (RV, $) of each type of vehicle in the fleet is modeled here as a percentage of the vehicle's MSRP

$$\mathrm{RV} = \eta \cdot \mathrm{MSRP} \quad \text{(Equation 46)}$$

The TCO of the BEV part of the fleet is thus computed

$$\begin{aligned}\mathrm{TCO}_{\mathrm{fleet,BEV}} &= \mathrm{IC}_{\mathrm{fleet,BEV}} + \mathrm{AOC}_{\mathrm{fleet,BEV}} \cdot \mathrm{YrsOp}_{\mathrm{fleet}} \\ &+ \mathrm{Penalty}_{\mathrm{dwell}} \cdot \mathrm{VMT}_{\mathrm{BEV,tot}} \cdot \mathrm{YrsOp}_{\mathrm{fleet}} \\ &+ \mathrm{Penalty}_{\mathrm{payload}} \cdot \mathrm{VMT}_{\mathrm{BEV,tot}} \cdot \mathrm{YrsOp}_{\mathrm{fleet}}\end{aligned} \quad \text{(Equation 47)}$$

The last two terms of the BEV TCO are related to the penalties associated with the dwell time and payload, which reflect challenges specific to early-stage electrification. Here, these are set as $\mathrm{Penalty}_{\mathrm{dwell}} = \$0.27/\mathrm{mile}$. We direct the reader to[23] for further details on the penalties. The TCO for the diesel part of the fleet is quantified by

$$\mathrm{TCO}_{\mathrm{fleet,diesel}} = \mathrm{IC}_{\mathrm{fleet,diesel}} + \mathrm{AOC}_{\mathrm{fleet,diesel}} \cdot \mathrm{YrsOp}_{\mathrm{fleet}} \quad \text{(Equation 48)}$$

The assumed TCO model variable values are obtained from a comprehensive TCO framework introduced in[23] for the electrified fleet, using real-world data provided by industry stakeholders, and are summarized in *Table S7*.

The cost to the fleet for is the ratio of the fleet TCO with the total vehicle miles travelled by the fleet over the years of operation,

$$\frac{\mathrm{TCO}}{\mathrm{mile}} = \frac{\mathrm{TCO}_{\mathrm{fleet,BEV}} + \mathrm{TCO}_{\mathrm{fleet,diesel}}}{\left(\mathrm{VMT}_{\mathrm{BEV,tot}} + \mathrm{VMT}_{\mathrm{diesel,tot}}\right) \cdot \mathrm{YrsOp}_{\mathrm{fleet}}} \qquad \text{(Equation 49)}$$

For comparison, we will compare the TCO/mile of the electrified fleet with the TCO/mile of a diesel-based HDCV fleet using the same framework above but with diesel rather than electricity as the fuel source. Assuming a fuel cost of $C_{\mathrm{fuel}} = \$0.68/\mathrm{mile}$, the other values listed in

Table S8, and performing the same cost analysis as above, we find that the cost of operating a diesel-based fleet is $1.20/mile.

5. Resource utilization to maximize air quality improvement in the Port of Savannah region

In this section, we examine how the microgrids, deployed at each the 171 viable sites identified by our proposed framework in Section 3, are dispatched to reduce the amount of pollutants generated if a solar and battery system are deployed at each site. We then examine the costs associated with deployment for each of the sites in the region.

5.1 Average utilization of resources

The average utilization of each resource is shown in *Figure S13* for the cases where: 1) only solar resources are built at each viable site and 2) where solar resources and a 100 MWh battery pack are deployed at each viable site. As expected, the average utilization of the grid decreases as sites with higher nameplate solar capacity are considered. As we saw in Section 5, the average utilization of solar generally remains the same even with the addition of a 100 MWh battery pack. Instead, the average utilization of the grid decreases, as the battery utilization increases. For the largest nameplate capacity site, we find that solar alone can only reduce the grid utilization to ~50%. While this still amounts to a significant pollutant intensity reduction, this illustrates the limitations of solar power in terms of efficiency and intermittency.

Additionally, in the case where a 100 MWh battery system is deployed, it is informative to examine the origin of energy used to charge the battery. Across all viable sites, greater than 80% of the time solar power is used to charge the battery; however, we observe that grid power is used to charge the battery for sites with lower nameplate capacities. This usage of the grid will incur a small amount of pollutant generation, contributing to the limited the air quality improvements for these lower nameplate capacity sites.

5.2 Capital costs of deployment

Using the capital costs estimates for both the solar and battery systems generated in Sections 7.1 and 7.2, we now estimate, for each viable site in the Port of Savannah, the cost of building a solar installation alone at the site, and a solar installation with a 100 MWh battery system collocated at that site. *Figure S15* illustrates that deploying a solar installation at sites with higher nameplate capacity will generally cost more, aligning with intuition. Moreover, co-locating a battery system with the solar installation will further increase the costs. For a given site, a lower total cost than the maximum observed here can be realized if a smaller size battery system or a smaller nameplate capacity solar farm is built at the site; however, this would also lower this site's pollutant mitigation capability.

5.3 Site-specific LCOPR

As we are considering the excess load demand for the full fleet, we consider the utility to be responsible in managing the costs of deployment of solar and battery resources. The fleet operator, then uses electricity from a grid that is augmented with these resources. We give

estimates of the costs for these two different stakeholders as quantified in Section 4 for every identified viable site in the Port of Savannah region.

The LCOPR for the viable sites in the Port of Savannah region are shown in *Figure S16*. We see a relatively stable value for the LCOPR of ~$26.40/MWh across the viable sites in the region, with only the lowest 5 nameplate capacity sites having a significantly higher LCOPR. The stable value, roughly speaking, results from the cost of larger nameplate solar capacity farms increasing proportionally to the energy output of the sites.

6. Capabilities of a solar site adjacent to the Port of Savannah

To systematically explore the effects of adding solar and energy storage into the energy network for a given site location, we explore the capabilities of the hypothetical microgrid deployment, incorporating solar and battery power, geographically adjacent to the Port of Savannah. The nameplate capacity of the solar farm and the battery size are assumed to be arbitrary. This hypothetical scenario does not account for the area requirements for deploying a microgrid and the land-use restrictions that would otherwise prevent a deployment directly adjacent to the port.

6.1 Available solar capacity estimation

To obtain an estimate of the available solar capacity $p_s(t)$ for the hypothetical site, we utilize the tool PVWatts by NREL[24]. This web application estimates the hourly available solar capacity, of a grid-scale photovoltaic system at a given location of the U.S. in a year. To do this, PVWatts cross-references the National Solar Radiation Database to determine which cell in the database corresponds to the desired location and uses that as an input to its energy generation prediction sub-models. To determine the hourly available solar capacity, we use PVWatts assuming a solar farm with a 1 MW nameplate solar capacity, where the solar panels are fixed, open-rack, with a 20-degree tilt and 180-degree azimuth angle. The default standard modules, corresponding to crystalline silicone cell material, is used. The system losses are estimated to be 14.08%. The available solar capacity profile is shown in *Figure S17*. To obtain the capacities for a larger nameplate capacity site, we linearly scale the 1 MW available solar capacity profile by the desired nameplate solar capacity.

6.2 Optimal air quality improvement under different scenarios

Using the available solar capacity estimate from PVWatts, we perform the dispatch optimization procedure described in Section 3 for each combination of nameplate solar capacity up to 100 MW and battery sizes up to 100 MWh and *Figure S18* shows the maximal total air quality improvement. In general, we find that the total air quality improvement increases monotonically both for increasing site nameplate solar capacity and battery size;

however, it is much more sensitive to the nameplate solar capacity. We observe that, for nameplate solar capacities of up to 100 MW and battery sizes up to 100 MWh, a maximum of ~36% air quality improvements reduction with the alternative energy network is achievable, corresponding to a site with 100 MW nameplate solar capacity and a 100 MWh battery system.

Notably, the contour lines in *Figure S18* are shallow with respect to battery size, indicating that the air quality improvement is relatively insensitive to the battery size. This arises due to the alignment of peak solar availability and excess load demand, as seen in *Figure S19* for the first five days of the year. The port uses the most electricity near noon time and attains the highest excess load demand at that time; coincidentally, noon time is also when solar availability is the highest.

The battery is expected to have a greater impact when the peak of the excess load demand is shifted with respect to the peak of solar availability. This scenario allows the battery to shift energy from periods of high solar availability to periods of high load demand. To explore this, we artificially shift the excess load demand signal ahead or delayed with respect to the peak of solar by 4 hours, as illustrated in *Figure S20*.

We find in the left and right subfigures of *Figure S21* that artificially shifting the excess load demand signal results in an increase in the sensitivity of the air quality improvement to the battery size, evidenced by the steeper slopes of the isolines with respect to the battery size. However, a demand shift also reduces the maximum air quality improvement attainable. For the maximum size of solar farm and battery system considered, the air quality can only be improved by ~30% when there is a demand shift present.

6.3 Resource utilization

To understand the limitations on the air quality improvement, we investigate the how the energy used meet the excess load demand is partitioned amongst the different energy sources. The average utilization of each resource is shown in *Figure S22* for different combinations of nameplate solar capacity and battery size, under different demand shift scenarios. Aligning with intuition, the average utilization of the grid decreases as sites with higher nameplate solar capacity are considered. Interestingly, for a given demand shift scenario, the average utilization of solar remains constant irrespective of the size of battery system added, as seen from the horizontal isolines in the solar utilization. As the battery system size is increased, for a fixed nameplate solar capacity, the average utilization of the grid decreases.

Interestingly, we observe that, even for sites with nameplate solar capacities of up to 100 MW and battery sizes of up to 100 MWh, it is impossible to meet the excess load demand

purely with solar and battery resources alone. The limited efficiency of solar resources imposes that grid resources must be used, leading to a limitation on the maximum air quality improvement.

Counterintuitively, due to the power limits on the battery, introducing a demand shift lowers the utilization of the battery for a given combination of nameplate solar capacity and battery size, relative to the no shift scenario. The power limitation of the battery is compensated for by grid power leading to the lower air quality improvement observed for the demand shifted scenarios.

6.4 Cost estimates of deployment

Having estimated the capabilities of a renewable energy network, where solar and battery system is deployed adjacent to the Port, we now estimate the cost of deployment for this system. We first focus on estimating the TEC, as it is the central quantity for the cost estimation for either of the stakeholders we consider in this work. To do this, we compute the LCOPR from the estimate of the hourly available solar capacity. We find that the LCOPR is constant with respect to nameplate solar capacity with a value of ~26 $/MWh. This indicates that the overnight capital costs scale linearly with the nameplate solar capacity of the hypothetical solar farm. Notably, the LCOPR is significantly lower than that of the grid electricity price of the Savannah region.

Given the average resource utilization computed from the optimal dispatch profiles of each network element, *Figure S23* shows the TEC under different demand shift scenarios and for different choices of LIB chemistry. For the NMC chemistry, we observe that the TEC isolines are relatively flat as a function of battery size. Instead, the TEC level for this chemistry seems most sensitive to the nameplate solar capacity. Additionally, due to the LCOPR being slightly lower than the grid electricity price, introducing a demand shift lowers the overall TEC as more solar power is used to meet the load demand. Using an LFP system can attain the lowest TEC out of all the other chemistries, particularly for larger nameplate solar capacities and battery system sizes. Due to its LCOS being much lower than the grid electricity price, a small increase in the battery system utilization for this chemistry can significantly decrease TEC. In contrast, as an NCA storage system has a significantly greater cost than the grid electricity price, an increase in the usage of the battery system causes an increase in the TEC. As such, the most cost-efficient system excludes an NCA battery system, regardless of the demand shift scenario. Computing the TEC allows us to subsequently compute the cost metrics for both the utility and the fleet operator. We will now examine each, in turn.

6.4.1 Cost to the utility

In *Figure S24*, a nonlinear relationship between system sizing and the total cost of ownership (TCO) per kilogram of pollutants removed is observed. Across all battery chemistries, the lowest TCO per kilogram of pollutants removed occurs at intermediate combinations of nameplate solar capacity and battery size. Undersized systems show higher costs due to insufficient ability to reduce pollutant intensity at lower solar and battery sizes, while oversized systems suffer from having higher maintenance costs. Demand-shifting strategies, such as advancing or delaying demand by four hours, consistently reduce the cost per kilogram of pollutants removed, highlighting the value of aligning energy use with renewable availability. Notably, LFP systems achieve lower minimum TCO values compared to NMC and NCA, underscoring the influence of battery chemistry on air quality improvement cost-effectiveness.

6.4.2 Cost to the fleet operator

The fleet operator cost metric is computed in *Figure S25* for different choices of battery chemistry and different demand shift scenarios. The TCO/mile trends closely mirror those observed for the TEC. In particular, the lowest cost battery chemistry is identified to be LFP, across all three demand shift scenarios. The NMC-based battery system is relatively insensitive to the battery system size, and the level of cost is largely determined by the nameplate capacity of the solar installation. An NCA-based battery system remains the most expensive and the lowest cost solution for this chemistry is a solar-only without an accompanying battery system.

We emphasize that the shape of the contours and the effect of the demand shift observed in the results of this section are specific to the Port of Savannah. As the hourly available solar capacity and pollution intensity are location dependent, if we chose a different location for our analysis the exact trends we would observe would vary. This highlights the importance of doing a region-based analysis to determine where a solar farm of a given size can viably be installed.

7. Site-specific utility cost metric

Site-specific utility cost metric

We show the estimated cost metric for the utility in

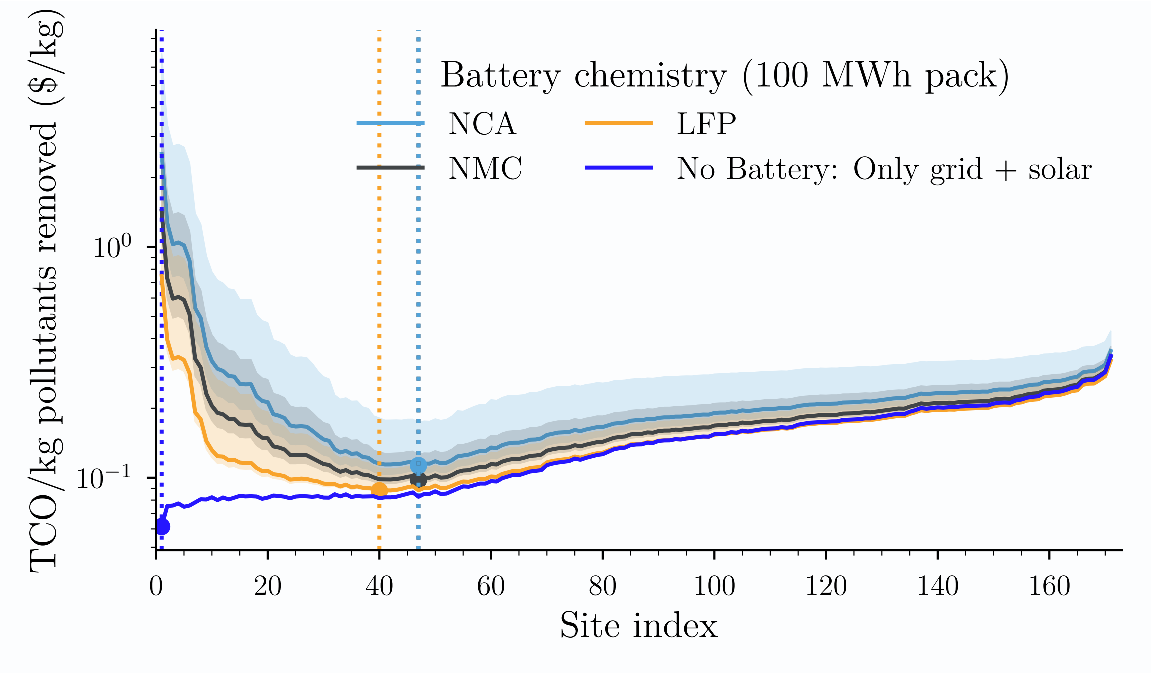

Figure *S26*. Notably, we find that there is a minimum in the cost metric at a site with an intermediate nameplate solar capacity when deploying the microgrid with a 100 MWh pack. On one hand, low nameplate capacity sites have a limited ability to reduce pollutant intensity but still must incur the capital costs of deploying solar and battery resources, leading to a high value of the utility cost metric. On the other hand, at high nameplate capacity sites due to the diminishing air quality gains fail to offset the rising deployment costs causing the cost metric to increase again. The competition between these two effects leads to a minimum in the cost for the utility.

The addition of a 100 MWh LFP battery system tends to decrease costs further, relative to when only solar is installed, beyond Site 55 and a 100 MWh NMC battery system beyond site 140. In contrast, the addition of a 100 MWh NCA battery system is always more expensive than the other options. For lower capacity sites, deploying solar alone results in a better cost metric. For these sites, there is insufficient solar availability to effectively utilize the battery system and thus the amount of air quality improvements realized by deploying a battery system cannot offset the increase in cost of deployment.

References

1. Sujan, V.A., Sun, R., and Snyder, I. (2025). OR-AGENT framework – Architecting electrified heavy-duty drayage applications. Applied Energy *386*, 125540. https://doi.org/10.1016/j.apenergy.2025.125540.

2. Moy, K. (2023). Towards Decarbonized Electric Grid and Transportation Sectors: A Modelling, Battery-Centric Study.

3. Annual Technology Baseline (2024). (National Renewable Energy Laboratory).

4. How Wind Can Help Us Breathe Easier Energy.gov. https://www.energy.gov/eere/wind/articles/how-wind-can-help-us-breathe-easier.

5. Denholm, P., Cole, W., and Blair, N. (2023). Moving Beyond 4-Hour Li-Ion Batteries: Challenges and Opportunities for Long(er)-Duration Energy Storage https://doi.org/10.2172/2000002.

6. Hou, X., Wang, J., Huang, T., Wang, T., and Wang, P. (2019). Smart Home Energy Management Optimization Method Considering Energy Storage and Electric Vehicle. IEEE Access *7*, 144010–144020. https://doi.org/10.1109/ACCESS.2019.2944878.

7. Klaas, A.-K., and Beck, H.-P. (2021). A MILP Model for Revenue Optimization of a Compressed Air Energy Storage Plant with Electrolysis. Energies *14*, 6803. https://doi.org/10.3390/en14206803.

8. Moradzadeh, M., and Abdelaziz, M.M.A. (2020). A New MILP Formulation for Renewables and Energy Storage Integration in Fast Charging Stations. IEEE Trans. Transp. Electrific. *6*, 181–198. https://doi.org/10.1109/TTE.2020.2974179.

9. Maclaurin, G., Grue, N., Lopez, A., Heimiller, D., Rossol, M., Buster, G., and Williams, T. (2021). The Renewable Energy Potential (reV) Model: A Geospatial Platform for Technical Potential and Supply Curve Modeling https://doi.org/10.2172/1563140.

10. Mays, G.T., Belles, R., Blevins, B.R., Hadley, S.W., Harrison, T.J., Jochem, W.C., Neish, B.S., Omitaomu, O.A., and Rose, A.N. (2012). Application of Spatial Data Modeling and Geographical Information Systems (GIS) for Identification of Potential Siting Options for Various Electrical Generation Sources https://doi.org/10.2172/1032036.

11. Omitaomu, O.A., Belles, R., Roberts, N., and Worrall, A. (2022). Methods and system for siting advanced nuclear reactors and evaluating energy policy concerns. Progress in Nuclear Energy *148*, 104197. https://doi.org/10.1016/j.pnucene.2022.104197.

12. Ramasamy, V., Zuboy, J., O'Shaughnessy, E., Feldman, D., Desai, J., Woodhouse, M., Basore, P., and Margolis, R. (2022). U.S. Solar Photovoltaic System and Energy Storage Cost Benchmarks, With Minimum Sustainable Price Analysis: Q1 2022. Renewable Energy.

13. Knehr, K., Kubal, J., Nelson, P., and Ahmed, S. (2022). Battery Performance and Cost Modeling for Electric-Drive Vehicles (A Manual for BatPaC v5.0) https://doi.org/10.2172/1877590.

14. Mauler, L., Duffner, F., Zeier, W.G., and Leker, J. (2021). Battery cost forecasting: a review of methods and results with an outlook to 2050. Energy Environ. Sci. *14*, 4712–4739. https://doi.org/10.1039/D1EE01530C.

15. Viswanathan, V., Mongird, K., Franks, R., Li, X., and Sprenkle, V. (2022). 2022 Grid Energy Storage Technology Cost and Performance Assessment.

16. Scorrano, M., Danielis, R., Pastore, S., Lughi, V., and Massi Pavan, A. (2020). Modeling the Total Cost of Ownership of an Electric Car Using a Residential Photovoltaic Generator and a Battery Storage Unit—An Italian Case Study. Energies *13*, 2584. https://doi.org/10.3390/en13102584.

17. Teichert, O., Link, S., Schneider, J., Wolff, S., and Lienkamp, M. (2023). Techno-economic cell selection for battery-electric long-haul trucks. eTransportation *16*, 100225. https://doi.org/10.1016/j.etran.2022.100225.

18. Average energy prices for the United States, regions, census divisions, and selected metropolitan areas https://www.bls.gov/regions/midwest/data/averageenergyprices_selectedareas_table.htm#ro5xgenergy.f.3.

19. Huang, S. (2022). Solar Energy Technologies Office Photovoltaics End-of -Life Action Plan https://doi.org/10.2172/1863490.

20. Gundogdu, B., and Gladwin, D.T. (2018). A Fast Battery Cycle Counting Method for Grid-Tied Battery Energy Storage System Subjected to Microcycles. In 2018 International Electrical Engineering Congress (iEECON) (IEEE), pp. 1–4. https://doi.org/10.1109/IEECON.2018.8712263.

21. Keil, P., Schuster, S.F., Wilhelm, J., Travi, J., Hauser, A., Karl, R.C., and Jossen, A. (2016). Calendar Aging of Lithium-Ion Batteries: I. Impact of the Graphite Anode on Capacity Fade. J. Electrochem. Soc. *163*, A1872–A1880. https://doi.org/10.1149/2.0411609jes.

22. Lam, V.N., Cui, X., Stroebl, F., Uppaluri, M., Onori, S., and Chueh, W.C. (2024). A decade of insights: Delving into calendar aging trends and implications. Joule, S2542435124005105. https://doi.org/10.1016/j.joule.2024.11.013.

23. Sun, R., Sujan, V.A., and Jatana, G. (2024). Systemic Decarbonization of Road Freight Transport: A Comprehensive Total Cost of Ownership Model. Preprint at arXiv, https://doi.org/10.48550/arXiv.2410.21026 https://doi.org/10.48550/arXiv.2410.21026.

24. Ryberg, D.S., Freeman, J., and Blair, N. (2015). Quantifying Interannual Variability for Photovoltaic Systems in PVWatts https://doi.org/10.2172/1226165.
